# A randomized controlled trial of a family-based HIV/STI prevention program for Black girls and male caregivers in Chicago: IMAGE study protocol paper

**DOI:** 10.1371/journal.pone.0320164

**Published:** 2025-03-28

**Authors:** Natasha Crooks, Geri Donenberg, Jessica Ogwumike, Jacqueline Silva, Ebere Udeogu, Emily Pela, Crystal Patil

**Affiliations:** 1 Department of Human Development Nursing Science, College of Nursing, University of Illinois Chicago, Chicago, Illinois, United States of America; 2 Center for Dissemination and Implementation Science, Department of Medicine, University of Illinois Chicago, Chicago, Illinois, United States of America; 3 University of North Carolina, Chapel Hill, North Carolina, United States of America; 4 Institute of Health Research and Policy, University of Illinois Chicago, Chicago, Illinois, United States of America; 5 School of Nursing, University of Michigan, Ann Arbor, Michigan, United States of America; PLOS: Public Library of Science, UNITED KINGDOM OF GREAT BRITAIN AND NORTHERN IRELAND

## Abstract

Black girls are disproportionately impacted by HIV and STIs in the United States. Black male caregivers are underutilized in family-based HIV/STI prevention programming and offer a novel opportunity to protect Black girls. This study will evaluate the efficacy and implementation of an HIV/STI prevention program delivered to Black girls and male caregivers in community settings. IMAGE: IMARA for the Male Caregivers and Girls Empowerment was adapted from IMARA, an evidence-based mother-daughter intervention to decrease HIV/STI infection and increase communication and healthy relationships among girls and their male caregivers. Using an efficacy implementation design, we will test the effectiveness of IMAGE compared to a time-matched general health promotion program across six community-based organizations. Aim one will evaluate IMAGE in a 2-arm randomized controlled trial with 300 14-18-year-old Black girls and their male caregiver dyads in Chicago, IL. We hypothesize that girls who receive IMAGE will have lower STI incidence (*primary outcome),* fewer sexual partners, and more consistent condom use (*secondary outcomes*) at 6- and 12-months compared to girls in the health promotion program. Guided by the 3-Step Implementation Model, our second aim is to identify and describe factors (barriers, facilitators) and processes affecting implementation in community settings. HIV and STI disparities go beyond individual-level factors, and male caregivers may protect girls by being a sexual health resource. This study will facilitate rapid CBO uptake and ownership of IMAGE if effective.

**Trial Registration:** ClinicalTrials.gov NCT06266416

## Introduction

Sexually transmitted infections (STIs) have been increasing disproportionately among Black girls and women in the United States (US). Among females aged 15–24 years, STIs are highest among Black girls and women across all US regions [1]. Rates of chlamydia among Black girls are five times the rate of white girls [1], further amplifying their risk for poor sexual and reproductive health (SRH) outcomes (e.g., pelvic inflammatory disease, infertility, HIV)[2]. In Chicago, STI rates are highest among 13–29-year-old Black females, and in 2018, 56% of new HIV diagnoses were among Black women [3]. During the COVID-19 pandemic, treatment of STIs dropped as new barriers to reproductive health care exacerbated existing challenges. The pandemic also illuminated high rates of intimate partner violence, a well-known correlate of HIV/STI [4].

Elevated HIV/STIs are associated with sexual violence [5], and estimates predict that one in every four Black girls will rates be sexually abused before the age of 18 [5,6]. During the COVID-19 pandemic, sexual education, mental health, and SRH services and curricula were nearly abandoned [7,8], exacerbating barriers to care and burdening individuals and their families with SRH education. Familial protection and family-based interventions may prevent exposure to sexual violence and STIs among Black girls while simultaneously strengthening family relationships and girls’ SRH [9–12]. *Becoming a Sexual Black Woman* is a framework that describes the sexual vulnerability and resilience of Black cisgender women during three developmental phases (Girl, Grown, and Woman) [13]. It details how sociocultural conditions exacerbate risk at each phase and how familial protection may prevent Black girls’ exposure to sexual violence and STIs [12,14]. Interventions that strengthen family relationships and communication align well with this framework and have demonstrated success in reducing sexual risk and STIs among Black girls [15,16]. For example, IMARA (Informed Motivated, Aware, and Responsible about AIDS) an evidence-based intervention for Black girls and their female caregivers, revealed a 43% reduction in STIs one year after the program ended [10].

Most family-based intervention programs focus on female caregivers (i.e., mothers, aunts, and sisters) and their role in the SRH of Black girls and young women [10,17,18]. Yet, Black male caregivers (BMC), inclusive of fathers, uncles, grandfathers, brothers, etc., often neglected in family-focused interventions, have a unique role in educating and protecting Black girls as they navigate understandings of sex, sexual violence, body image, and other topics considered taboo to discuss in a girl-male caregiver relationship [19–22]. Male caregiver involvement specifically might offer distinctive protection against sexual violence exposure during girls’ sexual development [23]. Evidence suggests that SRH programs that include male caregivers have been associated with later sexual debut and increased condom use in Black girls [19,22,24], as well as improved communication, relationship quality, and less condomless sex in boys [25–27]. Black girls want SRH information from male caregivers [23], and men want to be involved in protecting girls [28]. Engaging BMC in interventions may empower Black girls to reject unhealthy sexual advances that often lead to sexual violence.

IMAGE was developed to address the needs of Black girls and their male caregivers using the *Becoming a Sexual Black Woman* theoretical framework and the Health Disparities Research Framework (HDRF) [29]. The *Becoming a Sexual Black Woman* framework emphasizes the impact of structural determinants of health (racism, discrimination, sexual violence, stereotyped messages, and adultification) on Black girls’ sexual development pathways, sexual health, and decision-making. HDRF describes the influence of structural factors at the individual, interpersonal, community, and societal levels relevant to understanding and reducing health disparities [29]. Consistent with the HDRF framework, *Becoming a Sexual Black Woman* highlights how lack of protection is a form of structural violence that Black girls experience at individual, interpersonal, and societal levels. IMAGE leverages BMCs desire to participate in girls’ lives and protect and support them while challenging toxic masculinity and the use of dominance, violence, and control to assert power and superiority.

We followed the ADAPT-ITT framework’s eight steps to systematically tailor and adapt the IMARA curriculum to create IMAGE [30]. We conducted in-depth interviews with 30 Black male caregivers and six focus groups with Black girls, male caregivers, and female caregivers, and these data-informed curriculum refinements. Adaptations also addressed drivers of structural violence for Black girls (i.e., stereotype messaging, lack of protection). Following the adaptations, we “theater tested” IMAGE with six girl-male caregiver dyads (n = 12) in collaboration with a community organization on the West side of Chicago. By delivering IMAGE at community-based organizations, we strengthen the relevance for Black communities and the likelihood the program will be adopted and sustained.

IMAGE was revised based on feedback from the theater testing and then pilot-tested with 20 girl-BMC dyads. Findings revealed strong feasibility and acceptability: 87% of those approached agreed to participate; 93% completed the full intervention; 94% rated IMAGE as acceptable; and fidelity of intervention delivery was 98% [31]. Retention at one-month follow-up was 100%, and 87% agreed to return at 6-months. Preliminary data showed promising improvements in behavior and theoretical mediators.

### Community-based implementation

We partnered with community-based organizations (CBOs) to build community ownership IMAGE. We tailored IMAGE’s implementation to facilitate ease of community uptake. Based on the Exploration, Preparation, Implementation, and Sustainability (EPIS) framework [32]. We simplified EPIS into 3-Steps that CBOs can use to implement evidence-based interventions [33]. This approach has been successful for other behavior change interventions in clinical and community settings [33–36]. The 3-step approach supports CBO implementation and future ownership.

## Methods

### Design

This is a 2-arm randomized efficacy and implementation trial of IMAGE for 14-18-year-old Black girls and their male caregivers. Girl and BMC dyads will be assigned to workshops randomized to IMAGE versus a time-matched health promotion program (FUEL). All participants will complete baseline, 6- and 12-month follow-up assessments via REDCap. Girls will provide urine to screen for three STIs (trichomoniasis, chlamydia, and gonorrhea). IMAGE engages male caregivers in HIV/STI prevention for girls, thereby increasing its relevance to Black communities and the likelihood of adoption and sustainability by CBOs. Therefore, we will also assess implementation outcomes at CBOs in Chicago.

#### Aim 1 (Efficacy).

**Fig 1:**
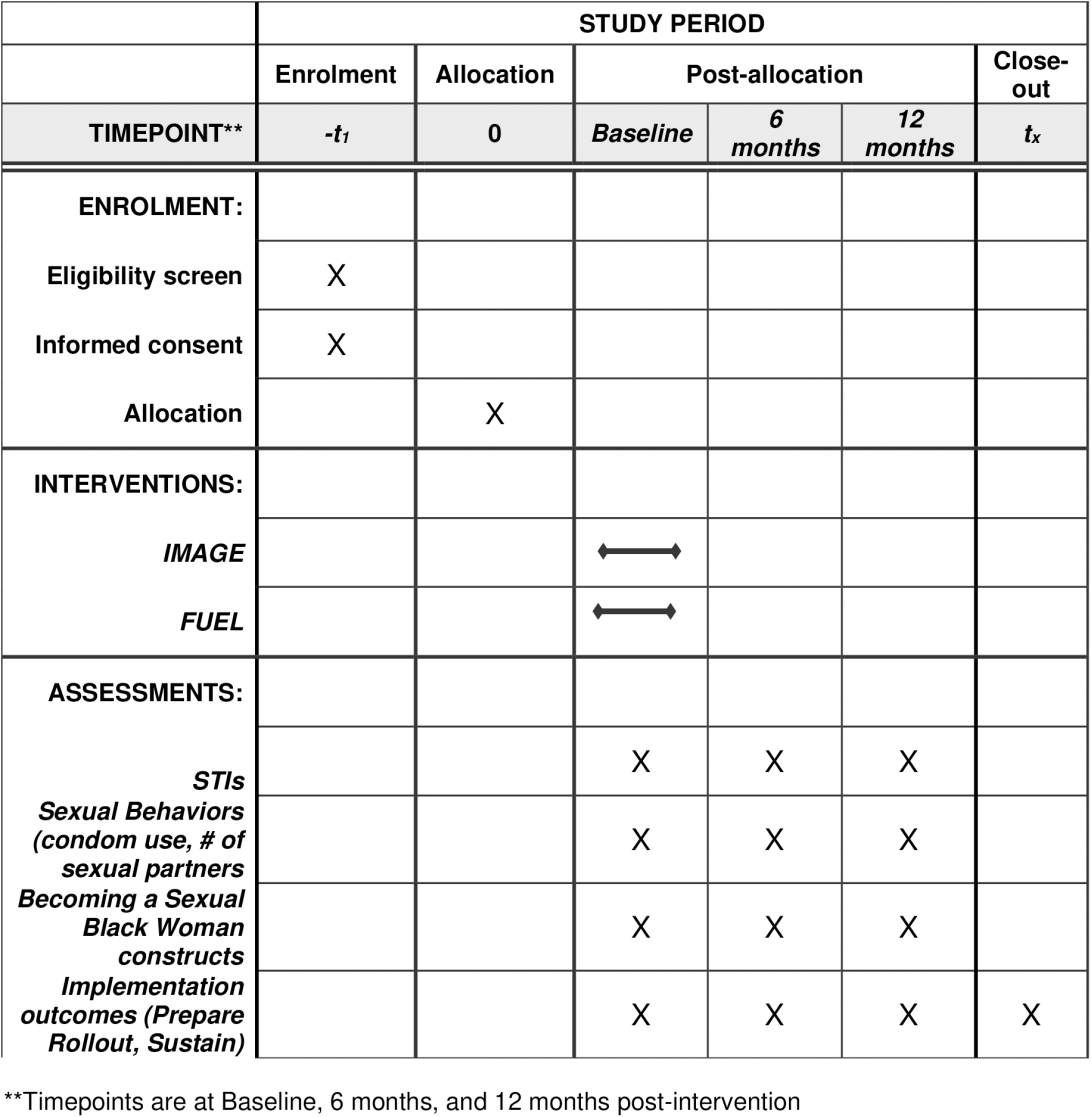
SPIRIT Schedule.

We will assign 300 14-18-year-old Black girls and their male caregivers to workshops randomized to either IMAGE or FUEL and assess girls’ STI incidence, sexual partners, and condom use at baseline, 6- and 12-months. We hypothesize that girls who receive IMAGE will have lower STI incidence (primary outcome), fewer sexual partners, and more consistent condom use (secondary outcomes). We will explore associations of individual, interpersonal, and structural factors proposed by the B*ecoming a Sexual Black Woman* and HDRF framework on primary and secondary outcomes at all three time points (Fig 1).

#### Aim 2 (Implementation).

We will identify and describe factors (barriers, facilitators, constraints) and implementation processes at six CBOs across Chicago.

#### Study setting.

We will deliver IMAGE at six CBOs on Chicago’s West and South sides impacted by structural factors (i.e., racism, poverty, crime, incarceration, and urban decay) implicated in high HIV and STI rates among Black communities. Neighborhoods in these areas are predominately Black, and Black females in these areas report the 4th highest rate of ever having sex (53%), the 3rd highest rate of sex before age 13, the 2nd highest rate of current sexual activity (40.6%) and have the highest rates of Chlamydia (1.921.0-2,688.4 per 100,000 people), and gonorrhea (973.7 - 1,536.2 per 100,000 people) [3]. Rates of sexual violence are also high in these neighborhoods [3]. The CBOs offer social services that address community resource needs and promote health and behavior change.

#### Aim 1 study population (efficacy).

Girls must be a) 14-18 years old; b) self-identify as African American, Black, or mixed race with African American or Black; c) speak English since measures are not normed for other languages; and d) identify an eligible male caregiver to participate in the study. BMC must be a) >  18 years old; b) self-identify as African American, Black, or mixed race with African American or Black; c) speak English since measures are not normed for other languages; d) be a current male caregiver to a Black girl 14-18 years old; and e) the girl’s legal guardian must agree to the male caregiver’s participation. BMC will be defined as men (fathers, grandfathers, uncles, brothers, cousins, etc.) who girls report playing a central role in their care and upbringing. Girls may or may not be sexually active, and information about girls’ sexual activity will not be shared with their male caregivers.

#### Recruitment, assent/consent, compensation and retention.

Recruitment will be multi-faceted. CBO will distribute flyers in the community and invite interested individuals to contact the IMAGE study. CBO will also contact eligible participants directly to inform them about the project and request permission for the research team to contact them. IMAGE staff will attend relevant community events to build relationships and conduct outreach. Using a screening and consent script, research staff will follow up with girls separately from guardians or male caregivers to conduct the screening, to obtain informed consent/assent with written, electronic signature via the e-consent module in REDCap, and to confirm the contact information of legal guardians and/or participating male caregivers. We will attempt to obtain girls’ assent/consent before we contact male caregivers, but participants may be screened in any order. Girls’ refusal to participate will precede consent or permission by male caregivers and legal guardians, regardless of the screening order. Dyads will complete surveys via REDCap at baseline, 6 and 12-month follow-up. Baseline survey assessments will occur before Day 1 of the intervention via REDCap. Girls and male caregivers will each receive $60 for completing the baseline assessment and an additional $65 for completing the entire workshop weekend. Participants are compensated $70 for the 6-month follow-up and $75 for the 12-month follow-up to recognize the value of retention over time. To overcome transportation barriers to STI treatment at the University of Illinois in Chicago Hospital Health Sciences System, we will provide an additional $5 in transportation compensation if needed. Participants’ transportation barriers will be assessed when scheduled for a workshop, and participant safety will be ensured. To maximize retention, we will use the strategies that were successful in our pilot (i.e., holiday cards and monthly phone calls or text messages) [31]. We will collect contact information for girls and male caregivers in multiple forms to find the most effective communication for each participant.

#### Randomization, sample size, and power.

We will use a stratified block randomization allocation to assign girl-BMC dyads to the treatment arm (IMAGE n = 150 or FUEL n = 150) prior to baseline assessment. Power analyses determined that a sample of 300 girls – 150 IMAGE and 150 FUEL is needed to assess efficacy on the primary outcome (STI incidence). Effect size (Cohen’s h) is a function of two proportions defined by Cohen; h = .2 is a small effect, h = .5 is a medium effect, and h = .8 is a large effect. We calculated the power to detect the effect of IMAGE vs. FUEL in reducing STI incidence as measured by having any of three STIs at 12 months post-intervention using a 2-sided test with alpha = .05. The new STI outcome at follow-up was a binary variable (yes/no to testing positive for at least 1 of the 3 STIs), because the study was not powered to examine specific STI outcomes. Based on the IMARA retention rate of 86% for Black female caregiver-girl dyads in Chicago [15], we evaluated the power of STI incidence (yes/no) at 80% retention over 12- months. Based on our previous work, we assume the STI incidence is 25% and 11% at 12 months for girls in the FUEL arm and IMAGE arm, respectively, which is a small-to-medium effect size of h = .37. With the proposed sample size, we will achieve 80% power to detect the intervention effect. We also calculated the standard effect size (Cohen’s d) for group mean comparisons with 80% power for our secondary continuous outcomes, where d = 0 is defined as no treatment effect. We can detect a small-to-medium effect size with the proposed sample size (d = .37).

#### Aim 1 efficacy study conditions.

**Intervention group: IMARA for black male caregivers and girls empowerment (IMAGE)**: IMAGE is an 8-10-hour HIV/STI group-based (6-8 dyads) prevention program delivered to girls and BMC, together and separated, reviewing parallel content. Modules address individual, social, and structural drivers of HIV risk (Table 1). The IMAGE curriculum was tailored specifically for Chicago’s Black girls and male caregivers. The topics discussed in the curriculum include the sexual development of Black girls, the risk of sexual violence, female anatomy, body positivity, HIV/STI knowledge and attitudes, and contraception use. IMAGE was created to foster healthy communication and connection between male caregivers and their girls by encouraging conflict resolution and perspective-taking. Male caregivers learn about the importance of mental health, role modeling, and partner choices. Through IMAGE, male caregivers will become more aware of their presence in their girls’ lives, discuss absenteeism (in the event they are incarcerated or unable to be physically present), and learn to be present. IMAGE emphasizes how Black girls and women are portrayed in the media, which creates unhealthy stereotypes, decreases self-image, and increases vulnerability to sexual violence. Girls and caregivers will discuss intimate partner violence, power dynamics, and healthy relationships, as well as discuss the use of contraceptives, specifically condoms. IMAGE directly addresses structural factors on girls’ HIV/STI risk, namely male incarceration, sexual violence, domestic violence, and negative stereotyping. Woven throughout IMAGE is the impact of mental health, gender-based violence, and alcohol and drug use on HIV risk.

**Table 1:** IMAGE Curriculum: Theoretical Constructs, Activities, and Target Participants.

Constructs	IMAGE Activities	Target
**Individual-level constructs**		
Sexual development	Becoming a Sexual Black Woman – theoretical framework overview	Joint
	Adolescent development jeopardy – developing body positivity and challenging assumptions	BMC Girls
	Birth control options resource guide and scenarios	Girls
HIV/STI knowledge, attitudes, beliefs and skills	Virus carrier handshake – assessing sexual partners’ risk behaviors	BMC Girls
	LIPSTICK – mnemonic acronym for condom use	BMC Girls
	Drugs and alcohol interactions with sex	Joint
	PrEP for Black women and female condom use	BMC Girls
	Keep It Simple Sister (KISS) – mnemonic acronym for responding to sexual pressure	Girls
	Guess Who – STI contraction, testing, and treatment	BMC Girls
Emotion regulation	Healthy coping with feelings as triggers for risk behavior	BMC Girls
	Destigmatizing mental health strategies for Black male caregivers	BMC
**Social-level constructs**		
BMC-Girl communication, conflict negotiation and effective communication	Role Plays – Passive, aggressive, and assertive communication	BMC Girls
	Role Plays – Different types of communication	Joint
	Discuss protection and navigating conflict between BMC-Girls	Joint
	Rephrase-it Game – Using I statements to communicate emotions	BMC Girls
Bonding, BMC as resources for HIV/STI prevention and protection	Getting to know one another – role plays to develop empathy	Joint
	Compliments for free – BMC-Girl bonding	Joint
	Discuss absenteeism, protection and incarceration	Joint
	The Big Talk – modeling BMC-Girl sex discussion	Joint
**Structural-level constructs**		
Stereotype endorsement and sexual objectification	Positive and negative portrayal of Black men and women in media	BMC
	Young, Black, and female – positive characteristics vs. stereotypes	BMC Girls
	Unpack toxic masculinity	BMC Girls
	Black female stereotypes (historical, media and cultural)	BMC Girls
Justification of violence in partner/relationship and partner dynamics	Gendered role stereotypes in relationships	BMC Girls
	Healthy versus unhealthy relationships, concurrent partnerships	BMC Girls
	Choosing healthy relationships, identifying abuse	BMC Girls
	Partner selection and implications for HIV risk	BMC Girls

BMC = Black male caregivers.

**Control condition: FUEL, a health promotion program**: We used a similar approach as described above (ADAPT-ITT) to tailor FUEL for Black girls and male caregivers in Chicago. FUEL promotes healthy living practices and topics, namely media literacy, healthy eating and physical activity, healthy eating and nutrition, physical activity, teen drug and alcohol use, and violence prevention (Table 2). Like IMAGE, topics are covered in both separate and joint sessions. FUEL has a brief HIV education module, but it does not address SRH dyad communication or conflict navigation. Video essays, documentaries, and information are used to address relevant topics.

**Table 2:** FUEL Curriculum: Health Promotion Topics.

Topics	FUEL Activities
Media literacy	Media’s influences body image, beauty, and strength
	Digital altering, filters, and social media trends’ impact on self-esteem
	Using personal collage to practice articulating healthier self-image
Healthy eating and physical activity	Combatting common missteps in diet/nutrition
	Benefits of exercise and meditation to overall health and well-being
	Planning physical activities that can be shared between BMC-Girls
Teen alcohol and drug use	Prevalence of drug and alcohol use among teens
	Impacts of drugs and alcohol on teen development
	Resources for harm reduction and smoking cessation
Violence prevention	Types and prevalence of violence (gang, domestic, bullying, dating)
	Localized examples of violence interruption and bystander intervention
	Discuss impacts of gun violence prevalence in Chicago

#### Intervention training.

Facilitators for both conditions must identify as Black, female, and have some experience working with Black communities or families. Facilitators were assigned to deliver IMAGE or FUEL. All facilitators completed 20 hours of training on their assigned curriculum and received supplemental one-hour training in-person and virtually to introduce key themes of cultural competency, PrEP, HIV prevention education, sex trafficking/human trafficking risk awareness, and general drug safety and navigating substance use disorders. Training for both programs stressed the importance of following the manualized protocol to ensure fidelity across facilitators and completing fidelity measurements at the end of each workshop. Following a curriculum review during the first two training sessions, facilitators practiced delivering each activity, taking turns serving as group leaders and participants. Each facilitator was “certified” as competent to deliver IMAGE or FUEL by the principal investigator and project director.

#### Aim 1 efficacy trial.

Six to eight dyads will be assigned to workshops randomized into FUEL or IMAGE at each CBO and will receive the corresponding curriculum on a designated weekend. Four facilitators and two observers are needed for each study condition; facilitators are not to be cross-trained in IMAGE and FUEL. Two facilitators co-lead the girls’ groups, and two co-lead the male caregivers’ groups. All four facilitators co-lead the joint sessions when girls and male caregivers come together for specific activities. Breakfast, lunch, and snacks are provided at all workshops for facilitators and participants.

#### Supervision, quality assurance, and treatment.

We will use a detailed manual and facilitator guide, conduct weekly supervision, and assess fidelity using facilitator self-report and observer ratings of adherence and competence (see below). Adherence measures determine if the program was delivered as intended (yes/no), and competence ratings indicate the quality of delivery. Each workshop is delivered by four facilitators and observed by two trained individuals to ensure fidelity and report on the quality of the workshop. Observers and facilitators will complete surveys after each workshop day, reflecting on the quality of facilitation. Participants will complete workshop evaluations. During ongoing supervision, the IMAGE research team will review fidelity reports and participant evaluations, discuss session activities and problem-solving difficulties, and provide feedback. If facilitators deviate from the curriculum, the supervisor will provide additional training until fidelity is achieved. We will complete observations of at least 40% of study workshops.

#### Contamination.

We will minimize contamination across arms. Facilitators will be assigned to IMAGE or FUEL and will not discuss or learn the other arm’s curriculum; training is done separately. The workshops will not be conducted on the same days; weekends will be divided, and each CBO will have only FUEL or IMAGE weekends to reduce participant contact. Confidentiality and a general orientation to the research structure were emphasized during facilitation training, allowing us to expect limited contamination.

### Intervention: 3-step sequence for implementation aim 2

We will utilize a 3-Step Implementation Model and its implementation guide [33] at each CBO to Prepare, Rollout, and equip them to Sustain IMAGE. The three steps are outlined more in the implementation procedures section.

### Step 1 prepare

Our team has previously developed partnerships with directors at six CBOs. The curriculum is designed to be flexible and capable of being delivered in various community settings. We will conduct site visits with each director, including environmental scans of prerequisite resources for hosting workshops. Each CBO director will appoint an internal liaison to lead recruitment activities and bridge communication between the sites and the IMAGE team, and the project director will interview liaisons to collect qualitative preparedness data. The information will be collected during site visits, interviews, and a brief prep phase survey to write a tailored implementation plan for each site per year. The implementation plan will then act as a living reference to guide workshop scheduling and execution for the year.

### Step 2 rollout

Each CBO hosts an equal number of IMAGE and FUEL workshops for the efficacy study (Aim 1).

### Step 3 sustain

With support and assistance from the implementation team, the CBO will review their experiences and decide if they will continue offering IMAGE at their location.

### Partnering CBO personnel

Each CBO follows the same sequence of steps to Prepare, Rollout, and Sustain IMAGE. The study will began with the Prepare phase, which involved engaging with the CBOs. During Rollout, the efficacy data (Aim 1) is collected across the 3 time points: baseline, 6, and 12 months. Aim 2 will formally evaluate and compare the implementation determinants (barriers, facilitators, constraints) and processes within and across six CBOs using data generated from surveys, observations, study notes, and interviews with CBO liaisons and directors, hereafter CBO personnel. *CBO personnel* (n = 12) will be asked and consented to participate in surveys and qualitative interviews. Each CBO director will select a liaison who is the reference contact for everything related to the workshops. CBO liaisons will oversee recruiting dyads and bridging the CBO and the IMAGE team. Inclusion criteria: All CBO directors (n = 6) and CBO IMAGE liaisons (n = 6) will be eligible. Because CBO liaisons will receive a stipend from UIC and are also CBO employees, we emphasize that participation in the interviews and surveys is completely voluntary. Personnel can refuse to participate without affecting their employment at the CBO or their stipend from UIC. Exclusion criteria: a) inability to understand the consent process, and b) non-employment at a partnering CBO. Regarding CBO personnel turnover (liaisons and/or directors), PI Crooks will engage new directors, and directors will appoint a new individual to serve as the liaison and be trained to perform their activities.

#### Aim 2 implementation procedures.

During ***Prepare***, we will conduct an environmental scan [37] at each CBO to describe and compare the inner and outer context across CBOs. We will identify structural and logistical resources (i.e., space, furniture, and other infrastructure). The scan also includes documenting current programming, the structure of decision-making, available resources (e.g., staff, space), and client engagement (e.g., number and frequency of the target population coming to the CBO). We review organizational charts to understand staff roles and determine how IMAGE fits the current structure. We identify the needs of each CBO and how the research team can support IMAGE delivery. We work with CBO directors to identify a liaison at the CBO who can interface with the community and assist in recruitment and implementation. We will share the materials related to IMAGE with the CBO personnel - director and liaison from each CBO (n = 12), and we will ask them to complete a survey [38] about the factors likely to affect IMAGE delivery and potential sustainability (ORIC and organizational climate measures). We will also conduct a 45-60-minute semi-structured interview with CBO liaisons (n = 6) involved in the IMAGE study (face-to-face, phone, or via Zoom) to document anticipated barriers and facilitators. The *Prepare* phase semi-structured interview only takes place with the liaison as a follow-up to the conversations held during the initial site visit with the CBO Directors. Verbal consent will be obtained before each interview. Participants will receive a $25 gift card for participating in the interview and survey. After the *Prepare* step, we will triangulate the data to co-create an adaptable implementation plan detailing how to deliver IMAGE at their site.

During IMAGE ***Rollout***, we will complete the Aim 1 efficacy study. After the first IMAGE session, we will dispatch a follow-up survey assessing implementation, adoption, and feasibility and conduct 30-45-minute semi-structured in-depth interviews (in person, by phone, or via Zoom) with CBO personnel (n = 12) to document facilitators, barriers and needed adaptations (e.g., to increase IMAGE enrollment and attendance). Verbal consent will be obtained before each interview, and participants will receive a $25 gift card.

After the trial ends (end of Rollout), CBO personnel will be asked to complete a third survey focused on acceptability, appropriateness, feasibility, and sustainability. We will conduct 45-60-minute semi-structured interviews (face-to-face, by phone, or via Zoom) with CBO personnel (n = 12) to assess each CBO’s total experience and capacity to ***Sustain*** IMAGE. Building on the environmental scan and previous interviews that identified facilitators, barriers, and solutions, we will ask CBO personnel to discuss their ability to integrate IMAGE into current programming. Verbal consent will be obtained before each interview. Participants will receive a $25 gift card.

## Study assessments

### Aim 1 measures

#### Primary outcome.

Black girls will receive STI testing at the CBOs on day 1 of their workshops. Trained research study staff will collect urine specimens from each girl at CBO sites. Urine specimens are delivered to the University of Illinois in Chicago Hospital Health Sciences System laboratory by a courier service, where trained clinical staff process the specimens for STI testing. The results of the STI testing are posted to EPIC, an electronic health records system. STI testing results are abstracted from EPIC and stored in REDCap. Trained research study staff call girls to communicate their STI testing results. Girls create a code word at the time of specimen collection. Research study staff are trained to confirm the name, date of birth, and the code word to ensure results are not shared with unauthorized parties.

#### Secondary outcomes.

Girls and male caregiver dyads will complete baseline, 6-and 12-month assessments via self-report surveys through REDCap. Sexual behavior will be measured by condom use and the number of partners. As a part of Aim 1, we will explore the three constructs of the *Becoming a Sexual Black Woman* framework*,* using self-report surveys that capture constructs of phases of sexual development, protection, and stereotyped messaging and measures that reflect all three levels of influence (individual-, interpersonal-, societal- levels) as outlined in the HDRF Framework (Table 3).

**Table 3:** Aim 1 Measures for Black Girls and Male Caregivers.

Becoming a Sexual Black Woman Framework Constructs	Operational Measure	Person*	
		G	BMC
**Phases of Sexual Development (Individual level)**			
Age of first intercourse	Age of first intercourse (vaginal, anal, and oral)	x	
Pubertal development	13-item scale describing perceived pubertal timing in comparison to peers, strategies to protect bodies and SRH, conditions influencing perceptions of bodies and behaviors and spaces feel most protected	x	
Condom self-efficacy	13-item measure of likelihood of condom use in diverse scenarios	x	
Sexual risk behavior	32-items, ARBA self-report of sexual behavior, assesses drug and alcohol use during sex (α = .79)	x	
HIV/STI knowledge, attitudes, and beliefs	18-item measure assessing factual information about HIV transmission, diagnosis, and prevention (α = .77)	x	x
Emotion regulation	20-item measure of emotion regulation, externally oriented thinking, and the ability to identify and describe feelings (α = 0.70)	x	
Coping with adverse life events	28-items self-report measures effective and ineffective ways to cope with stressful life events (α = .70)		x
Resilience	6-item, Brief resilience scale (α = .71)	x	x
Colorism	7 items, Skin Color Satisfaction Scale (α = .71)		
**Protection (Interpersonal level)**			
Caregiver-girl relationship and attachment	16-item scale, Child’s Report of Parent Behavior Inventory (CRPBI) perceived relationships of caregivers and girls	x	x
Sexual communication	20-item measure of perceived quality and quantity of risk-specific communication (e.g., having sex, condoms, and AIDS) (α=.65-.87)	x	x
Family risk and protection	38-item assessing 6 number of subscales on Family Conflict, Attitudes toward Attachment, Prosocial Involvement, Attitudes toward Drug Use (α = .66–.76)	x	x
Gendered racial socialization for BW	63- item, Gendered Racial-Ethnic Socialization Scale (GRESS) for Black women scale, gendered racial-ethnic socialization messages from caregivers regarding their identities (α = .67–.96)	x	
**Stereotyped Messaging (Societal level)**			
Stereotyping	11-item, to assess the extent to which participants endorse the sexualized stereotypes about Black women, to reflect the hypersexual and promiscuous nature of stereotypes. (α = 0.93)	x	x
Sexual objectification	11 item, Interpersonal Sexual Objectification Scale, experiences with having bodies evaluated (α = 0.91)	x	
Justification of Violence Scale	27-item, extent to which participants condoned IPV toward women when they behave in stereotype-consistent ways, (α = .99) for female and (α = .99) male sample	x	x
Everyday discrimination	5-item, Everyday Discrimination Scale, discrimination in everyday activities (α = .77)	x	x
G = Girls and BMC = Black male caregivers			

### Aim 2 implementation measures

Aim 2 data will be generated from the environmental scan, direct observations (fidelity ratings), study notes from meetings, semi-structured interviews documenting CBO personnel experiences hosting IMAGE (Table 4), and surveys (Table 5) of the CBOs’ attributes. Perceptions of acceptability, feasibility, and appropriateness of IMAGE will be assessed during *Preparation* after the *Rollout* phases, as well as views regarding IMAGE ownership during the *Sustain* phase.

**Table 4:** Aim 2 EPIS-Informed Semi-Structured Qualitative Interview Questions.

Outer Context
• What are your support structures/who supports your CBO (i.e., inter-organizational coalitions and relationships between entities, government, and funders)?
Inner Context
• How has the implementation of IMAGE been at your CBO? *Probe*: Did IMAGE address the needs of CBO families? • Tell me about your experience hosting IMAGE at your facility. *Probes*: How easy or difficult was it to run IMAGE at your CBO? Did IMAGE impact your work for the CBO? • What did you like and dislike about the IMAGE program? *Probes*: What were some of the inconveniences, difficulties, and problems you experienced while running IMAGE? Were you provided adequate support to host IMAGE? • What barriers do you foresee in running IMAGE at your CBO? *Probe*: What ideas do you have to overcome those barriers?
Bridging Factors
• How would you describe the relationship between you and the IMAGE team at UIC? *Probes*: What would you want to change about IMAGE? Do you feel like IMAGE aligns with your CBO mission?
Innovation
• At the completion of the study, would you want to continue offering IMAGE at your CBO, why or why not? • Is there anything else you would like to share about your experience with IMAGE? *Probe*: Is there a question you wish I’d asked during this interview?

**Table 5:** Aim 2 CBO Personnel Surveys.

Implementation phase	
Prep	**Organizational readiness.** Organizational Readiness for Implementing Change (ORIC) 12 items measuring confidence, commitment, motivation, and determination in implementing new interventions [39]
	**Organization climate.** CBO climate related to stress, workload, strain, and staff frustration [40]
Rollout	**Implementation, adoption and feasibility.** Inquiring about initial impressions of IMAGE programming after site first implements [41]
Sustain	**Appropriateness, acceptability, reach, and maintenance.** Assessing progressed impressions of IMAGE Programming at the end of efficacy trial [41]

#### Prepare.

We will conduct a baseline assessment at each CBO to describe the inner settings, bridging (CBO and UIC partnership), and innovation factors (IMAGE fit within each CBO). Aligning with implementation theories, we will ask CBO personnel to complete the Organizational Readiness for Implementing Change (ORIC) [39]. ORIC includes 12 items that measure confidence, commitment, motivation, and determination in implementing IMAGE. CBO personnel will also complete an Organizational climate [40] assessment, which consists of measures related to stress, workload, strain, and frustration, as these individual factors may influence the capacity to implement IMAGE in each setting.

#### Rollout.

Each CBO liaison will meet with the study’s project director weekly to discuss implementation challenges, recruitment, enrollment, rollout, and retention. Trained IMAGE facilitators will rate treatment fidelity of sessions when they act as observers. They will complete measures of adherence (delivery as planned) and competence (quality of delivery) for each session. The fidelity forms contain yes/no questions, including whether facilitators followed the session script/outline, explained and demonstrated each activity, provided corrective feedback, maintained an appropriate pace, and were open, non-judgmental, and comfortable with participants. The IMAGE research team will review fidelity checklists after each session. Areas of concern will be addressed with each facilitator during weekly supervision, and the facilitator(s) will be re-trained to reach fidelity as observed through our described model.

#### Acceptability, feasibility, and appropriateness.

We will document Black girls’ and caregivers’ *attendance* (Day 1 and Day 2) to indicate feasibility and acceptability. We will also select a random sample of five girls and five caregivers who complete IMAGE at each of the six CBOs (n = 60) to participate in qualitative interviews to document their experiences with IMAGE (each will be paid $50). Following consent/assent, we will ask about feasibility, acceptability, appropriateness, strategies to improve attendance, and reasons to deliver IMAGE in other venues. CBO personnel will complete the Acceptability of Intervention Measure (AIM) [42], Intervention Appropriateness Measure (IAM) [43], and Feasibility of Intervention Measure (FIM) [43], at the end of *Rollout.* The AIM assesses whether IMAGE is agreeable or satisfactory, the IAM assesses the perceived relevance or fits with each CBO’s mission, and the FIM captures the extent to which IMAGE was successfully carried out. Higher scores indicate greater acceptability, appropriateness, and feasibility.

#### Sustain.

The Program Sustainability Assessment Tool (PSAT) [44] will be used to evaluate potential IMAGE sustainability. The PSAT will generate a summary report of sustainability at the CBO and help inform sustainability planning. Individual scales represent organizational support, funding stability, positive academic and community-based partnerships, organizational capacity, program evaluation and adoption, stakeholder communication, and strategic planning (Fig 1).

## Data analysis

### Aim 1 analysis

#### Data reduction and preliminary analyses.

We will create summary scores for (1) Black girl-male caregiver relationship (quality and attachment), (2) Black girl-male caregiver communication (quality and quantity), and (3) girls’ risk behavior and attitudes (sexual behavior and condom use) [10]. We will create two separate forms of the dependent variable for girls: a composite score comprised of multiple risk indicators and a set of individual indicators (e.g., non-condom use, number of partners). We will compare groups on baseline variables using ANOVA (continuous) and Chi-square tests (categorical) and control for potential confounding variables in subsequent regression analyses that are not balanced by baseline randomization.

#### General statistical issues.

We will use Bonferroni correction to adjust for multiple comparisons as we will analyze multiple outcomes. We will control for Type I error in the analysis of the secondary outcomes but not the primary outcomes to capture important findings that may be obscured from the conservative limits imposed by Bonferroni correction. We will explore whether girls’ sexual behaviors are related to potentially important covariates (e.g., pubertal status) and use regression models to test their effects. To evaluate the impact of Black male caregivers on girls, we will include BMC’s characteristics as covariates in the regression model for girls’ outcomes. We will use a similar approach to evaluate the impact of girls on BMC over time. Missing data will most likely occur because of subject attrition. Based on our intervention studies, we expect 70%-85% retention in both arms. We will evaluate associations between dose (1 vs. 2 sessions) and efficacy. Missingness complicates statistical analyses via biased parameter estimates, reduced statistical power, and degraded confidence intervals. Based on our prior research, we expect minimal missingness in the data collection, but we will consider the item and unit nonresponse. An analysis using only completers is generally valid, assuming data are missing completely at random, but it is inefficient because it discards data points observed for non-completers. We will use all available data and produce valid inferences under the assumption that data are randomly missing. We will quantify the potential bias of these inferences should we suspect missing data to be non-random.

**Compare IMAGE vs. FUEL**^**TM**^ **on STI incidence and risky sexual behavior:** We will evaluate treatment effects separately at 6- months and 12 months. We will also combine data to evaluate an average effect across both time points and to analyze the change from 6- to 12 months. We will analyze binary outcomes using logistic and continuous outcomes using linear regression. At a single time point, we will test the effect of the binary indicator for IMAGE vs. FUEL on the outcome, adjusting for confounders and the outcome at baseline as additional independent variables. We will use mixed-effects regression models to analyze the outcome by combining both time points, and we will include a random intercept term to account for intra-subject correlation. We will test an average effect across time points and effects on patterns of change from 6- to 12 months by including an interaction term between treatment and time. We will use survival analysis to examine sexual initiation over the course of the study. We hypothesize that non-sexually experienced girls in IMAGE will, on average, initiate sex later than FUEL participants. We will estimate the Kaplan-Meier survival curves and conduct long-rank tests to compare the two survival curves. We will use Cox regression models and its extensions (e.g., Cox’s Proportional Hazard Model with Time-Dependent Covariates) to test for the effects of other covariates such as age or socioeconomic status.

#### Exploratory analysis.

Driven by the *Becoming a Sexual Black Woman* framework, we will use the regression-based causal mediation analysis approach [45] to test whether each of the individual, interpersonal, and structural (societal) level factors serve as a mediator of the intervention effect on the STI incidence, separately. Specifically, we will adopt the logistic regression model for the outcome and linear regression models for the mediators. We will report the controlled direct effect, natural direct and indirect effects, and proportion mediated with their 95% confidence intervals for each factor. We will adjust for sociodemographic characteristics collected at baseline as potential confounders in the outcome and mediator regression models. We will analyze sensitivity for unmeasured mediator-outcome confounding and report the mediational E-values [46]. We will also use a log-linear regression model for the outcome as a sensitivity analysis since our binary outcome may not be rare. We hypothesize that protection, represented by interpersonal factors such as dyad relationships, sexual communication, family risk, and protection (Table 3), would have the strongest mediation effects as it is most central to the *Becoming a Sexual Black Woman Framework.*

### Aim 2 mixed method data analysis

#### Quantitative analysis.

Survey data will be analyzed by our statistician using descriptive statistics (SAS, version 9.4), and an aggregated mean total score and means for each CBO will be calculated. Using a continuous and iterative process, we will identify contextual factors (events or statements) from observations (study notes and fidelity forms) and interviews to document what facilitates or acts as barriers to implementing IMAGE.

#### Qualitative analysis.

To begin the qualitative analysis, an RA and a study team member trained by the PI will immerse themselves in the data by reading and re-reading the transcripts and noting the interviewees’ perceptions. Using the Dedoose (Version 8.2.32) and following an approach described by Miles and Huberman [47], a set of initial codes grouped into broad domains reflecting the interview guide and EPIS framework (i.e., groups of related codes) will be developed. EPIS constructs will include Outer context – relationships between entities, including governments and funders; Inner context – the structure of CBOs, culture, networks, communication, climate, and readiness for implementation; and Bridging factors –the relationship between CBOs and UIC and Innovation – CBO and IMAGE fit and sustainability. The study team member will open code two transcripts simultaneously to refine codes into a preliminary codebook with clear operational definitions. Interviews will be separately coded, and then they will consult with the PI and other team members to review discrepancies, refine code definitions, and recode until intercoder reliability exceeds 85% [48]. Final codes will be compiled in the master codebook and applied to qualitative data coding. The research team will collaboratively analyze results, discuss codes, categories, and themes generated, and resolve discrepancies through discussions. This iterative process will allow us to identify the most salient contextual factors (events or statements) from observations (study notes) and interviews and document implementation barriers and facilitators (e.g., challenges, resolutions, impacts of champions, leadership, etc.). Final categories and themes will guide any necessary revisions of implementation procedures and the CBO’s implementation plan. We expect to identify shared and unique experiences from each CBO. We will triangulate qualitative data with quantitative measures related to treatment delivery and receipt of treatment. Together, these data will provide insight from all perspectives on program success and the potential future integration and sustainability of IMAGE by CBOs. Measures supporting rigor and trustworthiness in qualitative research include a detailed audit trail, study notes, and reflexivity notes. Each audio recording will be transcribed and checked for accuracy.

### Data management

Project protocols promote proper and timely data preparation for analysis and secure data storage. STI results, survey, and fidelity data will be collected in REDCap, with paper instruments used only in community settings without web access. All paper data will be entered into REDCap and destroyed once in the system. REDCap is a secure web-based data collection and management application for UIC faculty. The REDCap server is maintained by the Institute for Health Research and Policy staff at [blinded for review].

### Data monitoring

A trained data manager will monitor the data and review participant records, screening and consent documents, and data collection forms. We will assemble a Data Safety and Monitoring Board to review our activities to ensure participant safety and evaluate findings in an interim data analysis to determine if the RCT should continue or be stopped. Any protocol modifications or study amendments will be reported to the IRB.

### Adverse events

Adverse events related to the research are not expected. The facilitation role is curated to monitor potential study harm. The IMAGE team and staff will document and report any unanticipated harm to subjects within 24 hours to the PI and the IRB.

### Dissemination plan

**Fig 2:**
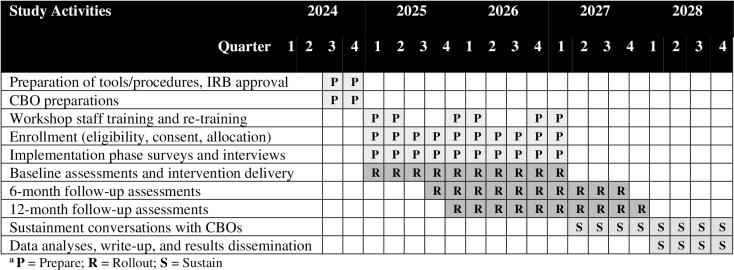
Study Timeline.

A series of publications will be published in the first year. In the second and third years, team members will identify areas of interest, generate outlines, and submit a qualitative paper. A grant writing workshop, led by the PI, will be held for our CBOs in year 4 to support sustainment in the future. In the 5th year, we will invite members of each CBO and other agencies involved in supporting Black families in Chicago to attend a workshop and share study results. Results shared in year five will allow organizations and policymakers to make informed decisions regarding IMAGE. Dissemination of this high-impact study is important because it will increase familial protective factors and reduce HIV/STI incidence for Black girls (Fig 2).

### Study status

When this manuscript was submitted for publication, the study was underway. All six partnering CBOs have completed the *Prepare* step. We’ve trained 16 facilitators and two supporting team staff members on FUEL and/or IMAGE. The first IMAGE workshop weekend took place in early October 2024. We have conducted four workshops at two CBO sites across Chicagoland. A total of 15 dyads are enrolled and/or awaiting research team contact.

## Discussion

This study will address a compelling need for innovative multilevel interventions to improve HIV/STI prevention for Black girls and lay the foundation for intervention sustainability in community settings. The study also fills a significant gap in HIV/STI programming for Black girls. Most family-based SRH interventions for Black girls exclude male caregivers due to structural factors that reduce opportunities for them to protect and support Black girls’ SRH. IMAGE offers a safe and structured environment for girls and male caregivers to develop, practice, and create effective communication skills that can be used in their relationships. IMAGE enhances BMC’s self-confidence in communicating with teen girls about sex and relationships while challenging societal norms and sexualized stereotypes that interfere with safe sex practices. We describe the design of an RCT of a family-based HIV prevention program, IMAGE, to reduce the incidence of HIV/STI in Chicago. The intervention draws on the *Becoming a Sexual Black Woman* and HDRF frameworks, which recognize multilevel influences of individual, interpersonal, community, and societal structural factors on Black girls HIV/STI risk and prevention. Prior to funding, IMAGE was extensively vetted with CBOs during theater and pilot testing and community advisory boards for approval. This process was essential to ensure study uptake and acceptability broadly and improve chances for sustainability.

We believe the study design has several strengths. We leverage the girl-male caregiver relationship to support HIV/STI prevention and protection of girls’ SRH health (bodies, behaviors, rejection of stereotypes). Furthermore, we believe that IMAGE is responsive to the age, gender, and cultural needs of Black girls and their male caregivers. This study is among the first to engage male caregivers in family-based SRH programming. By engaging male caregivers, this study leverages the unique strengths that male caregivers bring to the protection of girls. IMAGE targets two high-risk and vulnerable populations: Black girls and male caregivers. Black girls are particularly vulnerable to early sexual engagement, sexual violence, and HIV/STI infections, and Black males are disproportionately burdened by structural racism. The effects of individual-level SRH programs for Black girls decay over time. IMAGE may help sustain positive outcomes for girls as BMCs continue to deliver and reinforce prevention messages tailored to girls’ developmental phase after the formal intervention ends. IMAGE was designed to meet the standard for evidence-based interventions, advance intervention science with Black girls, and prepare for implementation and sustainability by engaging community stakeholders prior to the efficacy trial.

## Supporting information

S1 File Notice of Award from NIH. (PDF)

S2 File IRB approval. (PDF)

S3 File Study protocol. (DOCX)

S4 File SPIRIT checklist. (PDF)
