# Supplementary material for: A randomized controlled trial of a family-based HIV/STI prevention program for Black girls and male caregivers in Chicago: IMAGE study protocol paper
Source: PLoS One. 2025 Mar 28;20(3):e0320164. doi: 10.1371/journal.pone.0320164 (PMC11952266; doi:10.1371/journal.pone.0320164)
Supplement: S1 File — (PDF) [file pone.0320164.s001.pdf]

Notice of Award

FAIN# R01MD018929
Federal Award Date
09/24/2023

Recipient Information

1. Recipient Name
UNIVERSITY OF ILLINOIS
809 S MARSHFIELD AVE M/C 551
CHICAGO, IL 60612

2. Congressional District of Recipient
07

3. Payment System Identifier (ID)
1376000511A5

4. Employer Identification Number (EIN)
376000511

5. Data Universal Numbering System (DUNS)
098987217

6. Recipient's Unique Entity Identifier
W8XEAJDKMXH3

7. Project Director or Principal Investigator
Natasha Kaella Crooks, PHD

ncrooks@uic.edu
312-996-5801

8. Authorized Official
McCormack, Karen

312-996-2862

Federal Agency Information

9. Awarding Agency Contact Information
Sy Shackleford

NATIONAL INSTITUTE ON MINORITY HEALTH AND HEALTH DISPARITIES
shacklefords@mail.nih.gov
301-402-1366

10. Program Official Contact Information
Priscah Mujuru
Program Officer
NATIONAL INSTITUTE ON MINORITY HEALTH AND HEALTH DISPARITIES
priscah.mujuru@nih.gov
301-594-9765

Federal Award Information

11. Award Number
1R01MD018929-01

12. Unique Federal Award Identification Number (FAIN)
R01MD018929

13. Statutory Authority
42 USC 241 42 CFR 52

14. Federal Award Project Title
A Family-Based HIV Prevention Program for Black Men to Protect Black Girls

15. Assistance Listing Number
93.307

16. Assistance Listing Program Title
Minority Health and Health Disparities Research

17. Award Action Type
New Competing

18. Is the Award R&D?
Yes

Summary Federal Award Financial Information

**19. Budget Period Start Date** 09/25/2023 **– End Date** 05/31/2024	
**20. Total Amount of Federal Funds Obligated by this Action**	$730,541
20 a. Direct Cost Amount	$465,146
20 b. Indirect Cost Amount	$265,395
**21.** Authorized Carryover	
**22.** Offset	
**23.** Total Amount of Federal Funds Obligated this budget period	$730,541
**24. Total Approved Cost Sharing or Matching, where applicable**	$0
**25. Total Federal and Non-Federal Approved this Budget Period**	$730,541
**26. Project Period Start Date** 09/25/2023 **– End Date** 05/31/2028	
**27.** Total Amount of the Federal Award including Approved Cost Sharing or Matching this Project Period	$730,541

28. Authorized Treatment of Program Income
Additional Costs

29. Grants Management Officer - Signature
Priscilla Grant

30. Remarks
Acceptance of this award, including the "Terms and Conditions," is acknowledged by the recipient when funds are drawn down or otherwise requested from the grant payment system.

SECTION I – AWARD DATA – 1R01MD018929-01

Principal Investigator(s):
Natasha Kaella Crooks, PHD

Award e-mailed to: awards@uic.edu

Dear Authorized Official:

The National Institutes of Health hereby awards a grant in the amount of $730,541 (see "Award Calculation" in Section I and "Terms and Conditions" in Section III) to UNIVERSITY OF ILLINOIS AT CHICAGO in support of the above referenced project. This award is pursuant to the authority of 42 USC 241 42 CFR 52 and is subject to the requirements of this statute and regulation and of other referenced, incorporated or attached terms and conditions.

Acceptance of this award, including the "Terms and Conditions," is acknowledged by the recipient when funds are drawn down or otherwise requested from the grant payment system.

Each publication, press release, or other document about research supported by an NIH award must include an acknowledgment of NIH award support and a disclaimer such as "Research reported in this publication was supported by the National Institute On Minority Health And Health Disparities of the National Institutes of Health under Award Number R01MD018929. The content is solely the responsibility of the authors and does not necessarily represent the official views of the National Institutes of Health." Prior to issuing a press release concerning the outcome of this research, please notify the NIH awarding IC in advance to allow for coordination.

Award recipients must promote objectivity in research by establishing standards that provide a reasonable expectation that the design, conduct and reporting of research funded under NIH awards will be free from bias resulting from an Investigator's Financial Conflict of Interest (FCOI), in accordance with the 2011 revised regulation at 42 CFR Part 50 Subpart F. The Institution shall submit all FCOI reports to the NIH through the eRA Commons FCOI Module. The regulation does not apply to Phase I Small Business Innovative Research (SBIR) and Small Business Technology Transfer (STTR) awards. Consult the NIH website http://grants.nih.gov/grants/policy/coi/ for a link to the regulation and additional important information.

If you have any questions about this award, please direct questions to the Federal Agency contacts.

Sincerely yours,

Priscilla Grant
Grants Management Officer
NATIONAL INSTITUTE ON MINORITY HEALTH AND HEALTH DISPARITIES

Additional information follows

Cumulative Award Calculations for this Budget Period (U.S. Dollars)

**Salaries and Wages**	$280,552
**Fringe Benefits**	$92,267
**Personnel Costs (Subtotal)**	$372,819
**Consultant Services**	$11,600
**Materials & Supplies**	$14,100
**Travel**	$375
**Other**	$19,170
**Subawards/Consortium/Contractual Costs**	$41,310
**Tuition Remission**	$5,772

**Federal Direct Costs**	$465,146
**Federal F&A Costs**	$265,395
**Approved Budget**	$730,541
**Total Amount of Federal Funds Authorized (Federal Share)**	$730,541
**TOTAL FEDERAL AWARD AMOUNT**	$730,541
**AMOUNT OF THIS ACTION (FEDERAL SHARE)**	$730,541

**SUMMARY TOTALS FOR ALL YEARS** (for this Document Number)		
**YR**	**THIS AWARD**	**CUMULATIVE TOTALS**
1	$730,541	$730,541
2	$865,450	$865,450
3	$752,439	$752,439
4	$786,053	$786,053
5	$730,470	$730,470

Recommended future year total cost support, subject to the availability of funds and satisfactory progress of the project

Fiscal Information:

Payment System Identifier: 1376000511A5
Document Number: RMD018929A
PMS Account Type: P (Subaccount)
Fiscal Year: 2023

IC	CAN	2023	2024	2025	2026	2027
MD	8019793	$730,541	$865,450	$752,439	$786,053	$730,470

Recommended future year total cost support, subject to the availability of funds and satisfactory progress of the project

NIH Administrative Data:
PCC: CPS11 / **OC**: 41021 / **Released**: Grant, Priscilla 09/24/2023
Award Processed: 09/25/2023 12:05:55 AM

SECTION II – PAYMENT/HOTLINE INFORMATION – 1R01MD018929-01

For payment and HHS Office of Inspector General Hotline information, see the NIH Home Page at http://grants.nih.gov/grants/policy/awardconditions.htm

SECTION III – STANDARD TERMS AND CONDITIONS – 1R01MD018929-01

This award is based on the application submitted to, and as approved by, NIH on the above-titled project and is subject to the terms and conditions incorporated either directly or by reference in the following:

a. The grant program legislation and program regulation cited in this Notice of Award.
b. Conditions on activities and expenditure of funds in other statutory requirements, such as those included in appropriations acts.
c. 45 CFR Part 75.
d. National Policy Requirements and all other requirements described in the NIH Grants Policy Statement, including addenda in effect as of the beginning date of the budget period.
e. Federal Award Performance Goals: As required by the periodic report in the RPPR or in the final progress report when applicable.
f. This award notice, INCLUDING THE TERMS AND CONDITIONS CITED BELOW.

(See NIH Home Page at http://grants.nih.gov/grants/policy/awardconditions.htm for certain references cited above.)

Research and Development (R&D): All awards issued by the National Institutes of Health (NIH) meet the definition of "Research and Development" at 45 CFR Part§ 75.2**.** As such, auditees should identify NIH awards as part of the R&D cluster on the Schedule of Expenditures of Federal Awards (SEFA). The auditor should test NIH awards for compliance as instructed in Part V, Clusters of Programs. NIH recognizes that some awards may have another classification for purposes of indirect costs. The auditor is not required to report the disconnect (i.e., the award is classified as R&D for Federal Audit Requirement purposes but non-research for indirect cost rate purposes), unless the auditee is charging indirect costs at a rate other than the rate(s) specified in the award document(s).

This institution is a signatory to the Federal Demonstration Partnership (FDP) Phase VII Agreement which requires active institutional participation in new or ongoing FDP demonstrations and pilots.

An unobligated balance may be carried over into the next budget period without Grants Management Officer prior approval.

This grant is subject to Streamlined Noncompeting Award Procedures (SNAP).

This award is subject to the requirements of 2 CFR Part 25 for institutions to obtain a unique entity identifier (UEI) and maintain an active registration in the System for Award Management (SAM). Should a consortium/subaward be issued under this award, a UEI requirement must be included. See http://grants.nih.gov/grants/policy/awardconditions.htm for the full NIH award term implementing this requirement and other additional information.

This award has been assigned the Federal Award Identification Number (FAIN) R01MD018929. Recipients must document the assigned FAIN on each consortium/subaward issued under this award.

Based on the project period start date of this project, this award is likely subject to the Transparency Act subaward and executive compensation reporting requirement of 2 CFR Part 170. There are conditions that may exclude this award; see http://grants.nih.gov/grants/policy/awardconditions.htm for additional award applicability information.

In accordance with P.L. 110-161, compliance with the NIH Public Access Policy is now mandatory. For more information, see NOT-OD-08-033 and the Public Access website: http://publicaccess.nih.gov/.

This award provides support for one or more clinical trials. By law (Title VIII, Section 801 of Public Law 110-85), the "responsible party" must register "applicable clinical trials" on the ClinicalTrials.gov Protocol Registration System Information Website. NIH encourages registration of all trials whether required under the law or not. For more information, see http://grants.nih.gov/ClinicalTrials_fdaaa/

Recipients must administer the project in compliance with federal civil rights laws that prohibit discrimination on the basis of race, color, national origin, disability, age, and comply with applicable conscience protections. The recipient will comply with applicable laws that prohibit discrimination on the basis of sex, which includes discrimination on the basis of gender identity, sexual orientation, and pregnancy. Compliance with these laws requires taking reasonable steps to provide meaningful access to persons with limited English proficiency and providing programs that are accessible to and usable by persons with disabilities. The HHS Office for Civil Rights provides guidance on complying with civil rights laws enforced by HHS. See https://www.hhs.gov/civil-rights/for-providers/provider-obligations/index.html and https://www.hhs.gov/.

- Recipients of FFA must ensure that their programs are accessible to persons with limited English proficiency. For guidance on meeting the legal obligation to take reasonable steps to ensure meaningful access to programs or activities by limited English proficient individuals, see https://www.hhs.gov/civil-rights/for-individuals/special-topics/limited-english-proficiency/fact-sheet-guidance/index.html and https://www.lep.gov.
- For information on an institution's specific legal obligations for serving qualified individuals with disabilities, including providing program access, reasonable modifications, and to provide effective communication, see http://www.hhs.gov/ocr/civilrights/understanding/disability/index.html.
- HHS funded health and education programs must be administered in an environment free of sexual harassment; see https://www.hhs.gov/civil-rights/for-individuals/sex-discrimination/index.html. For information about NIH's commitment to supporting a safe and respectful work environment, who to contact with questions or concerns, and what NIH's expectations are for institutions and the individuals supported on NIH-funded awards, please see https://grants.nih.gov/grants/policy/harassment.htm.
- For guidance on administering programs in compliance with applicable federal religious nondiscrimination laws and applicable federal conscience protection and associated anti-discrimination laws, see https://www.hhs.gov/conscience/conscience-protections/index.html and https://www.hhs.gov/conscience/religious-freedom/index.html.

In accordance with the regulatory requirements provided at 45 CFR 75.113 and Appendix XII to 45 CFR Part 75, recipients that have currently active Federal grants, cooperative agreements, and procurement contracts with cumulative total value greater than $10,000,000 must report and maintain information in the System for Award Management (SAM) about civil, criminal, and administrative proceedings in connection with the award or performance of a Federal award that reached final disposition within the most recent five-year period. The recipient must also make semiannual disclosures regarding such proceedings. Proceedings information will be made publicly available in the designated integrity and performance system (currently the Federal Awardee Performance and Integrity Information System (FAPIIS)). Full reporting requirements and procedures are found in Appendix XII to 45 CFR Part 75. This term does not apply to NIH fellowships.

Treatment of Program Income:
Additional Costs

SECTION IV – MD SPECIFIC AWARD CONDITIONS – 1R01MD018929-01

Clinical Trial Indicator: Yes
This award supports one or more NIH-defined Clinical Trials. See the NIH Grants Policy Statement Section 1.2 for NIH definition of Clinical Trial.

RESTRICTION: Future year commitments are contingent upon the further review by the NIMHD of budget calculations. This award may be subject to reevaluation and

Version: 13 - 8/3/2022 12:59 PM | Generated on: 9/25/2023 12:05 AM

adjustment of the period of support and the funds awarded/committed depending upon the results of this review.

RESTRICTION: This award is issued without a currently valid certification of IRB approval for this project with the following special condition: Only activities that are clearly severable and independent from activities that involve human

subjects may be conducted under this award until the project has received IRB approval consistent with 45 CFR Part 46 and certification of IRB approval has been submitted to and accepted by the NIMHD in a revised Notice of Award. The certification should be sent via email to pg38h@nih.gov and shacklefords@mail.nih.gov no later than November 1st, 2023.

No funds may be drawn down from the payment system and no obligations may be made against Federal funds for research involving human subjects at any site engaged in such research for any period not covered by both (1) the recipient's OHRP-approved Assurance and if performance sites are involved, each performance site's OHRP-approved Assurance(s) and (2) appropriate IRB approvals consistent with all OHRP-approved Assurances. Failure to comply with this special condition can result in the suspension and/or termination of this award, withholding of support, audit disallowances, and/or other appropriate action.

RESTRICTION: This award is issued subject to the following special condition: In accordance with NIH policy, no research involving human subjects may be conducted on this project until the NIMHD receives certification that all key personnel as defined in the June 5, 2000 NIH Guide announcement, (revised August 25, 2000), "Required Education in the Protection of Human Subjects" (http://grants.nih.gov/grants/guide/notice-files/NOT-OD-00-039.html), have completed education on the protection of human subjects; and the NIMHD removes this restriction. The certification(s) for key personnel involved in this project, clearly identifying the grant number, must be sent no later than November 1st, 2023 to pg38h@nih.gov and shacklefords@mail.nih.gov. If the certification(s) is not received by the date listed above, this award may be suspended and may be terminated. The award also may be subject to audit disallowances and/or other appropriate action.

RESTRICTION: Funds awarded are contingent upon the receipt and acceptance by the NIMHD of current other support information. If this item is not received by November 1st, 2023 and depending on what it shows, this award may be subject to reevaluation and adjustment of the period of support and the funds awarded.

INFORMATION: Funds awarded are contingent upon the receipt and approval by the NIMHD of the grantee's response to the Initial Review Group's critique concerning the insufficient compensation for the intervention facilitators. It must be sent no later than November 1st, 2023 to pg38h@nih.gov and shacklefords@mail.nih.gov.

REQUIREMENT: This award is subject to the conditions set forth in PAR-21-358, Risk and Protective Factors of Family Health and Family Level Interventions (R01 - Clinical Trial Optional), NIH Guide to Grants and Contracts, 12/13/2021, which is hereby incorporated by reference as special terms and conditions of this award.

Copies of this RFA may be accessed at the following internet address: https://grants.nih.gov/grants/guide/pa-files/PAR-21-358.html

Copies may also be obtained from the Grants Management Contact indicated in the terms of award.

REQUIREMENT: The recipient is required to follow the data and safety monitoring plan included in the application and may not implement any changes in the plan without the written prior approval of the NIMHD.

REQUIREMENT: The recipient is required to follow the data sharing plan included in the application and may not implement any changes in the plan without the written prior approval of the NIMHD.

RESTRICTION: The clinical trial(s) supported by this award is subject to the plan submitted to NIH and the NIH policy on *Dissemination of NIH-Funded Clinical Trial Information.* The plan states that the clinical trial(s) funded by this award will be registered in ClinicalTrials.gov not later than 21 calendar days after enrollment of the first participant and primary summary results reported in ClinicalTrials.gov, not later than one year after the completion date. The reporting of summary results is required by this term of award even if the primary completion date occurs after the period of performance.

RESTRICTION: This award is subject to additional certification requirements with each submission of the Annual, Interim, and Final Research Performance Progress Report (RPPR). The recipient must agree to the following annual certification when submitting each RPPR. By submitting the RPPR, the AOR signifies compliance, as follows:

> In submitting this RPPR, the SO (or PD/PI with delegated authority), certifies to the best of his/her knowledge that, for all clinical trials funded under this NIH award, the recipient and all investigators conducting NIH-funded clinical trials are in compliance with the recipient's plan addressing compliance with the NIH Policy on Dissemination of NIH-Funded Clinical Trial Information. Any clinical trial funded in whole or in part under this award has been registered in ClinicalTrials.gov or will be registered not later than 21 calendar days after enrollment of the first participant. Summary results have been submitted to ClinicalTrials.gov or will be submitted not later than one year after the completion date, even if the completion date occurs after the period of performance.

INFORMATION: In order to redistribute awards more evenly throughout the year, budget periods are being adjusted. This award is issued with a shortened budget period and with 12 months of support. Continuation awards will cycle each year on June 1st.

Version: 13 - 8/3/2022 12:59 PM | Generated on: 9/25/2023 12:05 AM

INFORMATION: Although the budget period start date for this award is September 25th, this award includes funds for 12 months of support. Future year budget periods will cycle on June 1st. Allowable pre-award costs may be charged to this award, in accordance with the conditions outlined in the NIH Grants Policy Statement, and with institutional requirements for prior approval. The NIH GPS can be found on the internet at http://grants.nih.gov/grants/policy/nihgps/nihgps.pdf.

SPREADSHEET SUMMARY
AWARD NUMBER: 1R01MD018929-01

INSTITUTION: UNIVERSITY OF ILLINOIS AT CHICAGO

Budget	Year 1	Year 2	Year 3	Year 4	Year 5
Salaries and Wages	$280,552	$271,620	$284,796	$276,377	$286,686
Fringe Benefits	$92,267	$89,330	$93,663	$90,894	$94,284
Personnel Costs (Subtotal)	$372,819	$360,950	$378,459	$367,271	$380,970
Consultant Services	$11,600	$5,425	$5,000	$3,375	$6,000
Materials & Supplies	$14,100	$13,225	$3,425	$12,050	$2,150
Travel	$375	$2,000	$2,400	$2,950	$11,000
Other	$19,170	$130,200	$48,840	$76,500	$14,280
Subawards/Consortium/Contractual Costs	$41,310	$41,310	$41,310	$41,310	$41,310
Publication Costs			$3,000		$9,375
Tuition Remission	$5,772	$5,772	$5,772	$5,772	$11,544
TOTAL FEDERAL DC	$465,146	$558,882	$488,206	$509,228	$476,629
TOTAL FEDERAL F&A	$265,395	$306,568	$264,233	$276,825	$253,841
TOTAL COST	$730,541	$865,450	$752,439	$786,053	$730,470

Facilities and Administrative Costs	Year 1	Year 2	Year 3	Year 4	Year 5
F&A Cost Rate 1	59.9%	59.9%	59.9%	59.9%	59.9%
F&A Cost Base 1	$443,064	$511,800	$441,124	$462,146	$423,775
F&A Costs 1	$265,395	$306,568	$264,233	$276,825	$253,841
