# Supplementary material for: A randomized controlled trial of a family-based HIV/STI prevention program for Black girls and male caregivers in Chicago: IMAGE study protocol paper
Source: PLoS One. 2025 Mar 28;20(3):e0320164. doi: 10.1371/journal.pone.0320164 (PMC11952266; doi:10.1371/journal.pone.0320164)
Supplement: S2 File — (PDF) [file pone.0320164.s002.pdf]

IRB APPROVAL

January 2, 2024

Natasha Crooks
ncrooks@uic.edu

Dear Natasha Crooks:

On 12/19/2023, the IRB reviewed the following submission:

Type of Review:	Initial Study
Study Title:	A Family-Based HIV Prevention Program for Black Men to Protect Black Girls
Investigator:	Natasha Crooks
Study ID:	STUDY2023-1317
Expedited Review Categories:	6, 7
Funding:	Name: National Institutes of Health (Minority Health and Health Disparities), Grant Office ID: 115207, Funding Source ID: R01MD018929
IND, IDE, or HDE:	None
Documents Reviewed:	• Aim 2 Surveys v1.docx, Category: Subject Survey;<br>• CBO Interview Guides v1.docx, Category: Data Collection Tool;<br>• CH&A Letter of Support Crooks 09.19.23.pdf, Category: Letter of Support/Approval (non-UIC);<br>• Consent CBO v2.pdf, Category: Consent Form;<br>• Elite Empowerment_Crooks letter of support .pdf, Category: Letter of Support/Approval (non-UIC);<br>• FFL letter of support_6.8.23.pdf, Category: Letter of Support/Approval (non-UIC);<br>• LAMP_Partnership Letter__6.26.23.pdf, Category: Letter of Support/Approval (non-UIC);<br>• Letter of Support_First Tabernacle Beth EL Chicago.pdf, Category: Letter of Support/Approval (non-UIC);<br>• NOA, Category: Sponsor Attachment;<br>• Protocol v2.docx, Category: IRB Protocol;<br>• Recruitment Email v1.pdf, Category: Recruitment Materials;<br>• Register Interest v1.pdf, Category: Recruitment Materials;

The IRB approved the protocol on 12/19/2023.

To document consent, use the consent documents that were approved and stamped by the IRB. Go to the Documents tab to download them.

In conducting this protocol, you are required to follow the requirements listed in the Investigator Manual (HRP-103), which can be found by navigating to the IRB Library within the IRB system.

Sincerely,

Office for the Protection of Research Subjects
