# Supplementary material for: A randomized controlled trial of a family-based HIV/STI prevention program for Black girls and male caregivers in Chicago: IMAGE study protocol paper
Source: PLoS One. 2025 Mar 28;20(3):e0320164. doi: 10.1371/journal.pone.0320164 (PMC11952266; doi:10.1371/journal.pone.0320164)
Supplement: S3 File — (DOCX) [file pone.0320164.s003.docx]

**PROTOCOL TITLE:** A Family-Based HIV Prevention Program for Black Men to Protect Black Girls

**PRINCIPAL INVESTIGATOR:**

*Name:* *Natasha Crooks, PhD, RN*

*Department: Human Development Nursing Science*

*Telephone Number: 312-996-5801*

*Email Address:* [*ncrooks@uic.edu*](mailto:ncrooks@uic.edu)

**VERSION NUMBER/DATE:**

v15, 12/18/2024

**REVISION HISTORY**

| **Revision #** | **Version Date** | **Summary of Changes** | **Consent Change?** |
| --- | --- | --- | --- |
| 1 | 11/10/2023 | Initial submission |  |
| 2 | 12/11/2023 | Response to request for modification | Yes CBO v2 |
| 3 | 4/2/2024 | Adds Aim 1 workshop procedures | Yes Families v1 |
| 4 | 5/8/2024 | Adds Aim 1 workshop procedures – response to request for modifications | Yes Families v2 |
| 5 | 5/17/2024 | Adds Aim 1 workshop procedures – response to request for modifications | Yes Families v3 |
| 6 | 5/20/2024 | Adds Aim 1 workshop procedures – response to request for modifications | Yes Families v4 |
| 7 | 6/4/2024 | Removes references to the monitoring of self-assessment responses for risk of harm; these procedures were removed in a previous iteration of the protocol, as self-harm is not queried on the self assessments | No |
| 8 | 6/24/2024 | Updates and clarifies procedures related to STI Testing and Treatment, per requirements from UI Health and the CRFO. These changes concern:   1. the creation of a medical record in EPIC and the transmission of identifiers via the medical record to UI Health for all STI testing. 2. the addition of clinical procedures conducted outside the scope of the research by UI Health (i.e. pregnancy test and expedited partner treatment). | Yes |
| 9 | 7/20/2024 | Clarifies the function of MRNs as 1) an indirect identifier of urine specimens and 2) a link to STI testing and treatment results in the medical record; clarifies that MRNs, as identifiers, will never be exported with “coded data” from REDCap; adds language clarifying that certain sections of the Teen Assent/Consent form only apply to teens aged 18 years | Yes |
| 10 | 7/25/2024 | 1. Adds procedures to collect proxy contact information, our longstanding practice for longitudinal participant tracking and retention 2. Adds the date of birth to the labels and lab submitter forms that accompany urine specimens to the lab; this addition is made at the request of the UI Health Labs, to enhance accuracy and security, so that both research staff preparing specimens and lab staff processing specimens have a second identifier to cross-reference and confirm that the correct record is linked to the specimen 3. Updates the STI Notification Script to match previously approved protocol updates related to treatment and transportation to treatment offered to those teens who test positive for an STI 4. Adds an additional survey measure related to societal messaging and adultification | Yes |
| 11 | 8/15/2024 | 1. Adds script for reaching out to proxy contacts 2. Updates all documents to clarify that if a teen needs help getting a CTA pass, we will transmit $5 via electronic transfer to purchase one herself 3. Updates the Permission Form to add the name of the invited male caregiver, and submits a Permission Addendum, to align with previously approved procedures: “If the minor teen girl would like to invite a caregiver to participate who is not the guardian, UIC research staff will share the name of this person with the guardian when permission is obtained. If the teen girl later changes her mind about which caregiver she would like to invite, UIC research staff will re-contact the guardian for permission and will complete the addendum included in the permission form.” | Yes |
| 12 | 10/23/2024 | 1. adds additional survey measures related to legal history, skin color satisfaction, appearance and sexual decision-making, and resilience 2. updates the cadence of workshop reminder communications 3. adds language as requested by the CCTS to allow STI results to be transferred directly from EPIC into REDCap using the REDCap CDIS module; because results will be systematically transferred into REDCap from EPIC, they will no longer be verified by two separate staff members. 4. updates the workshop evaluation to be one consolidated measures for both caregivers and teen girls in both study arms   This modification does not alter any billable STI testing and treatment procedures and does not affect the current CRFO approved coverage analysis. | No |
| 13 | 11/13/2024 | 1. adds a CTA advertisement to recruitment activities.   This modification does not alter any billable STI testing and treatment procedures and does not affect the current CRFO approved coverage analysis. | No |
| 14 | 12/4/2024 | 1. Adds compensation for CBO recruitment staff based on the number of interested, eligible recruits 2. Adds the collection of social medial handles and mailing addresses for retention and follow up contact 3. Clarifies that we may consent girls either before or after we consent caregivers/guardians, even though the girls’ refusal will always take precedence over caregiver consent and guardian permission. 4. Adds additional STI testing for girls whose test results are negative but indeterminate 5. Updates screening and consent scripts (attached) to:    1. Remove and clarify redundant language    2. Make the tone more appropriate for teen girls    3. Clarify that the male caregiver cannot be a partner or boyfriend    4. Confirm whether participant has participated in an IMAGE workshop before   This modification may alter billable STI testing and treatment procedures and should be reviewed by CRFO for coverage analysis. | Yes |
| 15 | 12/18/24 | 1. Clarifies details around the collection of social media handles for retention | No |

Table of Contents

[1.0 Study Summary 2](#_Toc150354455)

[2.0 Objectives* 3](#_Toc150354456)

[3.0 Background* 3](#_Toc150354457)

[4.0 Study Endpoints* 3](#_Toc150354458)

[5.0 Study Intervention or Observation 3](#_Toc150354459)

[6.0 Procedures Involved* 3](#_Toc150354460)

[7.0 Data Collection and Retention* 3](#_Toc150354461)

[8.0 Sharing of Results with Subjects* 4](#_Toc150354462)

[9.0 Study Timelines* 4](#_Toc150354463)

[10.0 Subject Population* 4](#_Toc150354464)

[11.0 Vulnerable Populations* 4](#_Toc150354465)

[12.0 Number of Subjects 5](#_Toc150354466)

[13.0 Recruitment Methods 5](#_Toc150354467)

[14.0 Withdrawal of Subjects* 5](#_Toc150354468)

[15.0 Risks to Subjects* 5](#_Toc150354469)

[16.0 Potential Benefits to Subjects* 6](#_Toc150354470)

[17.0 Data Management* and Confidentiality 6](#_Toc150354471)

[18.0 Provisions to Protect the Privacy Interests of Subjects 6](#_Toc150354472)

[19.0 Economic Burden to Subjects 7](#_Toc150354473)

[20.0 Consent Process 7](#_Toc150354474)

[21.0 Setting 9](#_Toc150354475)

[22.0 Resources Available 9](#_Toc150354476)

# Study Summary

Sexually transmitted infections (STIs) continue to be a major public health problem for Black girls in the United States. Each year 1 in 4 Black girls, 14-19 years old acquires an STI, placing them at risk for poor sexual and reproductive health outcomes (SRH) (i.e., pelvic inflammatory disease, infertility, HIV/AIDS). In Chicago, STI rates are highest among 13- to 29-year-old Black girls, and they represent 56% of new HIV diagnoses compared to other racial groups, making adolescence an exceptionally vulnerable period. These racial disparities require new and innovative strategies to reduce Black girls’ negative SRH outcomes. Familial protection is seen as critical to mitigating risk, particularly exposure to sexual violence, which is linked to girls’ STI/HIV risk. Interventions that strengthen family relationships and communication as strategies to protect Black girls have demonstrated success in improving Black girls' SRH outcomes. Yet, with few exceptions, these programs engage only female caregivers, whereas male caregivers may amplify the protective effects of families on Black girls’ SRH. We systematically adapted IMARA (an evidence-based program designed for Black girls and their female caregivers) to create IMARA for Black Male caregivers and Girls Empowerment (IMAGE), adding drivers of structural violence (i.e., stereotype messaging and lack of protection) aligning with the Becoming a Sexual Black Woman framework and the Health Disparities Research Framework. Preliminary data (interviews, focus groups, theatre, and pilot testing) with Black girls and male and female caregivers justify the proposed randomized control trial (RCT). We will rigorously evaluate IMAGE’s efficacy in a randomized control trial while carefully documenting implementation determinants and processes to inform adoption and sustainability. Aim 1 is to conduct a 2-arm RCT (IMAGE vs. a health promotion control) with 300 14-18-yearold Black girls and their male caregivers and compare girls’ sexual risk behavior (condom use, sexual debut, and sexual partners) and STI incidence at baseline, 6- and 12-months. We hypothesize that girls in IMAGE will have lower STI incidence at 6- and 12- months (primary outcome) and report more condom use and fewer sexual partners (secondary outcomes) compared to the control group. We will also explore change in the theoretical mechanisms posited by the Becoming a Sexual Black Woman framework. Aim 2 is to identify processes, barriers, and constraints associated with primary outcomes to inform future sustainability in community-based organizations. The long-term significance and impact of this application are high. By including Black male caregivers in the protection of girls, this study leverages a long-neglected yet important resource in Black girls' SRH, thereby amplifying the protective effects of family-based programs and pushing the science of health disparities forward.

# Objectives*

Aim 1. Compare SRH outcomes at 6- and 12-months in 14-18-year-old teen girls (n=300) randomized to IMAGE or a time-matched control program. Hypothesis: Girls who receive IMAGE will have lower STI incidence (primary outcome), fewer sexual partners, and more consistent condom use (secondary outcomes). Exploratory analysis: We will explore associations of individual, interpersonal, and structural factors proposed by the Becoming a Sexual Black Woman framework on primary and secondary outcomes at 6- and 12 months.

Aim 2. Describe implementation factors and processes across community-based organizations (CBO) using mixed methods. Each CBO will use the 3-Step Implementation Model, and through mixed methods, we will identify and describe factors (i.e., barriers, facilitators, constraints) and processes affecting implementation at each of the five CBOs.

# Background*

Disparities in STIs/HIV are a health crisis in Black communities in the United States.^39,40^ In 2018, Black individuals accounted for 42% of new HIV diagnoses, and 11% were among Black women.^41^ In Chicago, STI rates are 2-3 times the national average and highest among 13-29 year-old Black females. ^2,41,42^ Each year 1 in 4 Black girls 14-19 years old acquires a STI, increasing their risk for long-term poor sexual and reproductive health outcomes (SRH) (e.g., pelvic inflammatory disease, infertility, and HIV).^1^ Sexual violence, a driver of STIs/HIV acquisition, is also a grave concern in Black communities; 20% of U.S. Black women are raped during their lifetime,^40^ and 25% of Black girls will be sexually abused before age 18.^3^ The COVID-19 pandemic exacerbated rates of sexual violence and STIs, in part, by eliminating school-based sex education and decreasing access to mental health care and SRH services.^5^

This project is guided by the Becoming a Sexual Black Woman framework,^6^ which is informed by the Health Disparities Research Framework (HDRF).^43^ Many theories of behavior (e.g., social cognitive, self-efficacy, and planned behavior^37,38,44–61^) fail to address structural factors or the dyadic nature of social interactions that drive sex and sexual violence, especially for adolescents.^62,63^ By contrast, the Becoming a Sexual Black Woman framework is culturally relevant, developmentally tailored, and grounded in the lived experiences of Black girls and women. It highlights the impact of structural factors on the sociocultural context of Black girls and women.^6–9^ Published by Crooks et al.,^6^ the Becoming a Sexual Black Woman framework is the result of five years of qualitative inquiry^6–9,64^ that describes sociocultural processes and conditions influencing Black female sexual development, namely stereotyped messaging and familial protection.^8^ We aligned the constructs of the Becoming a Sexual Black Woman framework with the Health Disparities Research Framework (HDRF),^43^ which describes the influence of structural factors at the Individual, Interpersonal, Community, and Societal **
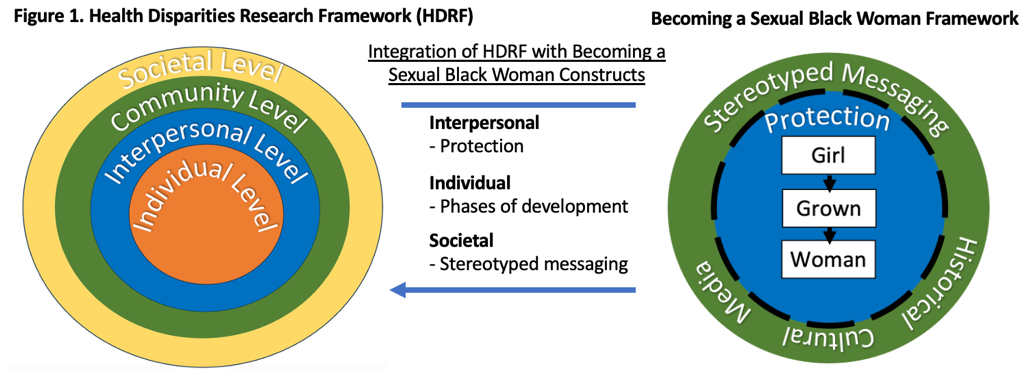
**levels relevant to understanding and reducing health disparities (see Figure 1). Becoming a Sexual Black Woman framework emphasizes the impact of structural determinants of health (racism, discrimination, sexual violence, stereotyped messages, and adultification) by reducing the protection of Black girls’ sexual development pathways, sexual health, and decision-making.^43^ It delineates three phases of Black female sexual development (Girl, Grown, and Woman). Movement across the phases depends on the level of Protection experienced and the Stereotyped messaging present. Protection is conceptualized as a means to prevent early sexual engagement, sexualization, and sexual violence of Black girls.^8^ Historically, Black female caregivers have offered protective strategies (i.e., sharing of stories, communication about sex and relationships) to Black girls about SRH focused on their bodies, sex, and relationships.^8,20,65–67^ Stereotyped messaging is defined as visual images, words, or stories in social media, culture, and history that sexualize Black female bodies and promote sexual behavior.^6^ Black girls are particularly vulnerable to historically (i.e., Jezebel)^68,69^ and culturally (i.e., fast or fast-tailed girl)^7,70^ rooted sexualized stereotyped messaging, leading to body shame and silence about sexual violence.^6^ These messages justify and normalize sexual violence against Black girls and women. Social media exacerbates the risk of sexual violence and STI/HIV by perpetuating sexual objectification of Black bodies.^8,71^ In our research, Black girls reported that parental protection mitigated the effects of stereotyped messaging on negative sexual health outcomes and expressed a desire for the same protections from Black male caregivers.^7^ Consistent with the HDRF framework, Becoming a Sexual Black Woman specifically highlights lack of protection as a form of structural violence Black girls experience at individual, interpersonal, and societal levels.

The scientific premise of this research is that individual, interpersonal, and structural factors impact Black girls’ SRH outcomes (STI/HIV) and experience of sexual violence. The proposed study expands STI/HIV prevention programs to include Black male caregivers, a potentially valuable yet underutilized resource to protect Black girls and reduce their exposure to STI/HIV and sexual violence.  Individual, interpersonal, and structural factors drive Black girls’ vulnerability to sexual violence and STIs.^43^ At the individual level, Black girls start puberty around 8-9 years old while white girls begin around 12-14 years old.^72,73,74^ Early pubertal development increases risk for sexual abuse, STIs,^6,75,76^ early sexual debut,^73,74,77,78^ ^79^ and sexual contact before 13 years old.^7,72,80^ Black girls who look older than their peers get labeled as “fast” or promiscuous.^7,70,72^ At an interpersonal level, early pubertal maturation can cause adultification of Black girls (i.e., being treated and viewed as adult women) and lack of protection during childhood.^8,65,75^ ^75,81^ Adultification inappropriately exposes girls to adult knowledge and assigns them adult roles and responsibilities.^76^ At a structural (societal) level, Black girls are stereotyped as hypersexual and experience elevated rates of sexual violence.^7,79^ The prevalence of violence against Black women is likely underestimated, as Black girls may not recognize their first sexual encounters as violent or assaults and do not report them.^82^ Sexual violence creates feelings of shame and is linked to high-risk sexual behavior.^79^ A key structural factor driving Black girls’ sexual health outcomes is the harm committed against Black men as a result of inequitable social, political, and economic factors that reduce opportunities to protect and support their children.^9,10^ Disproportionate rates of incarceration, police brutality, and lack of economic prospects contribute to the myth and misconception of the “absent Black father” stereotype.^83^ Yet, research shows that Black male caregivers (i.e., fathers, grandfathers, uncles, brothers, cousins) are present in girls’ lives^84^. Interventions involving Black male caregivers and Black boys^23,85,86^ show improved communication and relationship quality, help boys avoid violence and remain safe,^87^ and protect against condomless sex among boys.^13,14,25,21–23^ Black male caregivers are viewed as credible and trustworthy messengers about safety and protection among boys because of their similar exposure to structural racism.^87^

Black male caregivers may also be uniquely positioned to mitigate the impact of individual, interpersonal, and structural factors driving STIs and sexual violence in Black girls. The presence of Black male caregivers is associated with later sexual debut and increased condom use among female adolescents,^13,14,21,88^ but male caregivers have been largely ignored as potential partners in STI/HIV prevention for Black girls,^25^ especially in relation to sexual violence prevention. Black girls want to receive SRH information from male caregivers noting their unique perspectives as “men”.^16,17,24^ Historically, Black male caregivers have been prescribed distinct gendered social roles as provider and protector, making them ideal collaborators in programs to reinforce protective factors for Black girls SRH and sexual violence.^25^ Single fathers are a growing public research priority, increasing from 1.7 million in 1990 to 3.3 million in 2020.^89^ For Black girls, it is understood that they face unique challenges in identity development and that Black female caregivers are typically tasked with preparing them to encounter racism and sexism.^90^ However, Black male caregivers have developed their own protective strategies (i.e., utilizing their own racial experiences, developing awareness of discrimination and coping, and cultivating strong positive personal and cultural identities) that they can impart to Black girls to amplify their protection.^91^ Black male caregivers can promote the sexual health of Black girls through gendered-racial socialization, which has been identified in the literature as a protective factor in Black girls' sexual behavior.^65,92–95^ Interventions that strengthen male caregivers’ comfort communicating about sexual health and practical skills to promote safer sex behaviors with Black girls may be the missing link to strengthen positive outcomes of family-based programs.^16,17,24,25^

Several programs have demonstrated positive effects on Black girls’ sexual behavior and STIs,^15,36,37,96^   but ongoing disparities in STI/HIV infections underscore the need for innovative approaches.^64^ One evidence-based intervention is well-positioned to engage Black male caregivers and serve as a foundation for a Black male caregivers-girl program. IMARA (Informed, Motivated, Aware, and Responsible Adolescents and Adults; formerly “about AIDS”), a psychosocial STI/HIV prevention program designed for Black girls and their female caregivers, addresses individual, interpersonal, and structural drivers of STI/HIV risk.^15^ IMARA leverages the female caregiver-girl dyad as a structural resource for girls to encourage behavior change, in part by strengthening girls’ perceptions of female caregivers as a resource in sexual decision-making and by shifting peer norms within the group in favor of prevention. The curriculum seeks to strengthen female caregiver-girl relationships and communication, enhance self-efficacy to use condoms, teach assertive communication, increase maternal monitoring, improve emotion regulation, and help families recognize the role of social media and stereotyped messaging on Black girls, all while emphasizing pride in Black culture and gender empowerment. IMARA is one of the only family-based programs that focuses on the parent-adolescent relationship related to SRH and empowers parents as role models. It promotes Black values and underscores the impact of social and cultural drivers related to STI/HIV risk. IMARA can be adapted to include additional structural factors (e.g., sexual violence, male incarceration).^97^ In a 2-arm RCT, girls who received IMARA demonstrated a 43% reduction in STI incidence at 12-month follow-up compared to girls who received a time-matched family-based health promotion program.^15^ By engaging diverse stakeholders and employing rigorous methods, we adapted IMARA for Black male caregivers in Black girls SRH to create IMAGE.^97^

IMAGE (IMARA for Male Caregivers and Girls Empowerment). In collaboration with Chicago North Lawndale Amachi Mentoring Program (LAMP), a CBO in Chicago’s North Lawndale community, we used the ADAPT-ITT framework^30^ to create IMAGE for Black girls and male caregivers. IMAGE emphasizes Black male caregivers' desire to participate in girls’ lives, and protect and support them while challenging toxic masculinity,^98^ and the use of dominance, violence, and control to assert power and superiority. Modules focus on sexual violence and objectification of Black girls and women, relationships, and how to be a positive role model for Black girls. IMAGE is delivered over 2-days which has several advantages over multi-session interventions: (1) It maximizes participation so dyads can benefit from the full program. (2) Two days ease the burden on families' financial resources. (3) Two sessions have real-life utility; they are pragmatic and responsive to the needs of urban low-income families. (4) Research supports psychosocial and behavioral change among girls following brief HIV prevention interventions.^15,99^ IMAGE engages Black male caregivers and single-father homes in STI/HIV prevention for girls, thereby increasing its relevance to Black communities and the likelihood of adoption and sustainability by community-based organizations.

Community engagement is essential to this family-based HIV intervention addressing a major public health concern. To move quickly along the translational continuum,^100^ we bring in implementation science at this early stage. Our goal is to facilitate rapid community-based organization (CBO) uptake and ownership of IMAGE, should it result in positive outcomes. The Exploration, Preparation, Implementation, and Sustainability (EPIS) framework^29^ describes four implementation phases and delineates the influences of outer (policies, funding, cross-agency cooperation) and inner (organizational capacity, climate, leadership) contexts on implementation processes. It also includes bridging factors such as the nature of the intervention and the role of innovation. EPIS has successfully guided the implementation of services for sexual health for youth and HV/STI prevention^101,102^ and mental health,^17,150,151^ which justifies our selection of this framework. Together with the bridging (CBO and UIC partnership) and innovation factors (IMAGE fit within each CBO), we draw on climate and organizational readiness theories and measures to explore how these aspects of the CBO relate to interconnections, interactions, linkages, and relationships. EPIS guided our adaptation of IMARA into IMAGE^97^ (Exploration and Preparation). With our partner CBO, we addressed barriers to implementation (Exploration), planned delivery (e.g., trained staff, hired the CBO liaison) (Preparation), and piloted IMAGE (Implementation) with 40 dyads with high fidelity. For this proposal, we use a 3-Step Implementation Model that simplifies EPIS into an accessible tool that community organizations can use; this has been successfully used for other behavior change interventions in clinical and community settings.^31,103–105^ The 3-Step Implementation Model and its implementation guide^31^ incorporates perspectives from diverse stakeholders to position each CBO to meet their mission, empower communities, and increase the likelihood of uptake and sustainability.^106^ This pragmatic implementation strategy supports CBO implementation and future ownership by building implementation capacities and describing the step-by-step procedures for collaboration between academic partners and CBOs as we Prepare, Rollout, and potentially Sustain IMAGE.  We will describe how organizational climate,^107^ readiness for change,^108^ and the attributes of each CBO relate to implementation, which will strengthen CBO capacity for future ownership of this evidence-based intervention.

# Study Endpoints*

Aim 1: Primary outcomes include sexual and reproductive health outcomes (STI incidence, self-reported sexual behavior) at 6- and 12-months among 14-18 year old teen girls (n=300) randomized to IMAGE or a time-matched general health promotion control program (FUEL). We will explore the three constructs of the Becoming a Sexual Black Woman framework, using self-report surveys that capture constructs of phases of sexual development, protection, and stereotyped messaging and measures that reflect all three levels of influence (individual-, interpersonal-, societal- levels) as outlined in the Health Disparities Research Framework.

Aim 2: Secondary outcomes will be assessed via data collection from CBO personnel and include:

- The Organizational Readiness for Implementing Change (ORIC), a 12-item measure of confidence, commitment, motivation, and determination in implementing IMAGE,
- a measure of Organizational Climate, related to stress, workload, strain, and frustration, as these individual factors may influence the capacity to implement IMAGE in each setting, and
- measures to assess the adoption, maintenance, acceptability, appropriateness, feasibility, and sustainability of IMAGE.

# Study Intervention or Observation

In collaboration with Chicago North Lawndale Amachi Mentoring Program (LAMP), a CBO in Chicago’s North Lawndale community, we used the ADAPT-ITT framework^30^ to create IMAGE (IMARA for Male Caregivers and Girls Empowerment) for Black teen girls and male caregivers.

IMAGE is an 8-10-hour HIV/STI group-based (6-8 dyads) prevention program delivered to Black male caregivers and teen girls over 2-days. IMAGE is delivered by trained Black female facilitators to improve teen girls’ SRH outcomes and reduce sexual violence. Over the two days, some components of the curriculum are delivered separately to male caregivers and teen girls, covering parallel content, and other sections are delivered jointly in a single group. The curriculum, extensively tailored for the target population and pilot tested, addresses Black teen girls' sexual development, risk for sexual violence, female anatomy, body positivity, HIV/STI knowledge and attitudes, and condom use. IMAGE is designed to strengthen bonds and communication between male caregivers and teen girls by encouraging perspective-taking (i.e., reverse role play) and conflict resolution. Male caregivers learn about the importance of mental health, role modeling, and partner choices on their ability to protect Black teen girls, create monitoring plans to increase engagement in teen girls’ lives, and discuss absenteeism (when they cannot be physically present) in the event they are incarcerated or cannot be physically present to protect teen girls. IMAGE underscores how current/popular images of Black teen girls and women reinforce unhealthy stereotypes and the impact of social media (i.e., TikTok and Snapchat) on teen girls’ self-image and vulnerability to sexual violence. Separately, teen girls and caregivers discuss intimate partner violence and the value of healthy relationships (see Table 2). Interactive learning is used to support skill-building (condom use), and IMAGE directly addresses structural factors on teen girls’ STI/HIV risk, namely male incarceration, sexual violence, domestic violence, and negative stereotyping.

IMAGE emphasizes Black male caregivers' desire to participate in teen girls’ lives, and protect and support them while challenging toxic masculinity,^98^ and the use of dominance, violence, and control to assert power and superiority. Modules focus on sexual violence and objectification of Black teen girls and women, relationships, and how to be a positive role model for Black teen girls. IMAGE is delivered over 2-days which has several advantages over multi-session interventions: (1) It maximizes participation so dyads can benefit from the full program. (2) Two days ease the burden on families' financial resources. (3) Two sessions have real-life utility; they are pragmatic and responsive to the needs of urban low-income families. (4) Research supports psychosocial and behavioral change among teen girls following brief HIV prevention interventions.^15,99^ IMAGE engages Black male caregivers and single-father homes in STI/HIV prevention for teen girls, thereby increasing its relevance to Black communities and the likelihood of adoption and sustainability by community-based organizations.

Time-matched control program–FUEL. FUEL, used in prior research, is a caregiver/adolescent general health promotion program identical in length and intensity to IMAGE. FUEL will engage Black male caregivers and teen girls to promote good nutrition, exercise, and informed consumer behavior. Topics include the impact of media on body image, evaluating nutritional labels to make healthy food choices, eating balanced meals, establishing regular exercise routines, and how families and communities can support healthy behavior. FUEL includes a brief video about HIV/AIDS and other STIs but otherwise does not otherwise address sexual health. Like IMAGE, FUEL is delivered in groups of 6-8 dyads over two workshop days (~10 hours total) in one weekend. Parts of the curriculum are delivered separately to teen girls and male caregivers covering parallel content and other components are delivered jointly.

# Procedures Involved*

- 1. The current protocol seeks approval for both study aims 1 and 2.

Study Aim 1 is a 2-arm RCT (IMAGE vs. a health promotion control) with 300 14-18-year-old Black teen girls and their male caregivers to compare teen girls’ sexual risk behavior (condom use, sexual debut, and sexual partners) and STI incidence at baseline, 6- and 12-months.

Study Aim 2 is a non-randomized, mixed methods evaluation of barriers, facilitators, and constraints to the implementation of IMAGE within CBOs. CBO directors and liaisons will be invited to participate in interviews and REDCap surveys to share their perspectives as professionals within the CBO.

- 1. **Aim 1 procedures in chronological order** are as follows:
     1. UIC research staff and/or CBO staff will conduct a variety of outreach and recruitment activities, including:
     - posting recruitment flyers at the CBOs or other community sites.
     - sharing recruitment flyers via social media platforms, websites, or listservs. Social media platforms will only be used for passive recruitment, directing interested participants to the REDCap Registration Form (see 6.2.2) – social media will not be used to directly interact with interested participants.
     - approaching families in person to share the flyer, using the Approach Script.
     - distributing flyers at relevant outreach events held either in person or in presentations over video conferencing platforms (e.g., Juneteenth, Bold expressions festivals, community events, etc.) – Flyers will be shared via Zoom using visual presentation of the flyer on a shared screen.
     - distributing flyers to participants who complete workshops, encouraging them to tell other families about the program.
     - posting paid advertisements on buses and trains in the Chicago Transit Authority (CTA) system, geographically targeted to the neighborhoods where CBOs are located; ads contain the same QR codes & language as previously approved recruitment flyers (please see IMAGE CTA Ad attached).

If interested participants request that CBO staff give their contact information to UIC researchers, CBO staff will transmit contact information to the UIC research team via the Registration Form (see #2). UIC research staff will only call families who have entered their contact information in the Registration Form. CBO staff (themselves research participants within Aim 2) will be involved in the implementation of IMAGE at their organizations, as it is hoped they will continue to run the IMAGE program after research activities have concluded. CBO staff will therefore be involved in the delivery of IMAGE for administrative non-research purposes only, i.e., conducting outreach to encourage participation in workshops and coordinating on-site logistics (e.g. supervising the use of the site by unlocking the doors or organizing meal service, etc.), but they will not participate in or be present in the room during the delivery of the workshop materials. Furthermore, CBO staff will **not** be involved with any research procedures, i.e., screening, consent, assessment, STI testing, or retention procedures; and they will not have access to research records or REDCap. All research procedures will be conducted by UIC staff.

- - 1. CBO staff are either directly employed by or affiliated with the community-based organizations who host workshops. All CBO staff, both employees and affiliates, will follow recruitment procedures as stated above. CBO staff may be compensated for their recruitment efforts as described above. To avoid any undue influence or coercion on recruits to participate, CBO staff will receive a fixed $ amount for each interested and eligible teen girl recruited, regardless of whether the girl decides to participate in the study or not. Interested participants can either 1) call/text/email research staff directly using contact information on the flyer/ad or 2) access a QR code on the flyer/ad that links to a secure REDCap form (see REDCap Registration Form) where they can enter their name, phone number, email address, and preferred time of contact. Caregivers who complete the REDCap registration form will also be asked to provide the name and contact information for a teen girl they think might be interested in participating. Teen girls who complete the form will be asked to provide the name and contact information of a caregiver they might want to invite to participate, as well as their legal guardian. The REDCap registration form will state that a parent or guardian’s permission is required for minors (14-17 years).
    2. Using the Screening and Consent Script, research staff will follow up with teen girl research candidates separately from guardians or caregivers to conduct the screening, to obtain consent/assent with written electronic signature via the e-consent module in REDCap, and to confirm the contact information of legal guardians and/or participating caregivers. We will attempt to obtain teen girls’ assent/consent before we contact caregivers, but participants may be screened in any order. Teen girls’ refusal to participate will take precedence over consent or permission by male caregivers and legal guardians, regardless of the screening order
    3. Research staff (not CBO staff) will follow up with caregivers and/or guardians as appropriate to obtain consent and/or permission with written electronic signature via the e-consent module in REDCap. If the minor teen girl would like to invite a caregiver to participate who is not the guardian, UIC research staff will share the name of this person with the guardian when permission is obtained. If the teen girl later changes her mind about which caregiver she would like to invite, UIC research staff will re-contact the guardian for permission and will complete the addendum included in the permission form. For an invited male caregiver who is also the legal guardian of a teen girl, we will obtain both consent for himself to participate and permission for the teen girl to participate.
    4. Research staff may conduct screening and consent procedures by phone, via UIC Zoom, or in person in a private space. All consent procedures will be conducted via REDCap, even if conducted in person. Links to consent forms in REDCap will either be emailed or texted to the participant, according to their preference, or study tablets or laptops may be used if consent is conducted in person. No paper forms will be used. Staff will confirm the study was explained fully, the study participant could understand the information provided, and the study participant was given ample opportunity to ask questions. REDCap will be configured to automatically send signed consent/assent/permission forms to participants via email or text according to their preference. No study-related procedures will be completed prior to obtaining informed consent.
    5. After agreeing to participate, participants will be scheduled to attend an upcoming workshop and asked to provide social media handles where they can be reached via private direct message, a mailing address, and the name and phone number of someone (e.g., a family member or friend) who we can contact if we can’t reach them over the course of the study. Staff will restrict social media communications to the following: “Hi, this is [name] from the UIC IMAGE study. We are trying to get in touch with you! Please call or text us at (872) 260-6648, email us at image@uic.edu, or let us know a number where we can reach you.” Staff will inform participants that we will keep our social media direct message communication vague, and that social media sites collect information regarding online activities, as per the usage agreement participants accepted to use the site, and may share this information with others, including advertisers.
    6. On or around the Monday before a scheduled workshop, or if after that Monday, as soon as a participant has been enrolled to participate in a workshop scheduled the following Saturday, research staff will send participants secure REDCap links to baseline self-assessment surveys via email or text message. Self-assessment surveys should take about an hour to complete.
    7. On or around the Wednesday before a scheduled workshop, research staff will send workshop reminders via call, text, or email to participants who have not confirmed attendance or completed baseline self-assessment surveys.
    8. On or around the Friday before a scheduled workshop, research staff will send workshop reminders via call, text, or email to all participants.
    9. STI TESTING:

This research offers teen participants (not caregivers) STI testing using biological endpoints (yes/no). STI testing involves two separate components: 1) UIC research staff will obtain urine specimens from teen girls ; and 2) Specimens will be transported to the UI Health Laboratory and screened for three sexually transmitted pathogens: N. gonorrhoeae, C. trachomatis, and T.vaginalis. If teen participants receive an indeterminate test result, they will be contacted to re-test at a community partner site or similar convenient location.

UIC research staff will generate a medical record for each teen participant in EPIC, if they do not already have one. The following information will be added to the record: the participant’s name, date of birth, and telephone number. When generating a medical record in EPIC, a medical record number (MRN) will be generated and assigned to the teen participant.

On the morning of workshop day 1, UIC research staff will provide teen girls with collection containers and escort them to a private, secure room in which to produce the urine specimen. UIC research staff will decant urine specimens to centrifuge tubes labeled with the MRN and date of birth and store the tubes in a baggie with a paper lab submitter form (a form which is required by the UI Health Laboratory). The paper lab submitter form includes the participant’s MRN and date of birth and tells the UI Health Laboratory which tests to perform on the sample.

The baggies with the specimens will be stored in a secure lock box provided by the UI Health Laboratory. Only the courier service arranged by the UI Health Laboratory will have access to the secure lock box using a key. If a secure lock box from the UI Health Laboratory is unavailable, the specimens will be stored in a locked cooler or similar locked container, which only the UIC study team will have access to. The locked cooler/container will remain in the custody of the study team at all times until picked up by the courier service or otherwise transported to the laboratory.

The courier service will pick up the samples from the community-based organization the same day they are collected. Tracking features enable the samples to be tracked by the UI Health laboratory to ensure secure delivery to the UI Health lab. If a courier service is unavailable, a UIC study staff member will transport the samples to the UI Health Laboratory the same day they have been collected. Once the samples are at the UI Health laboratory, a trained tech or manager within the UI Health laboratory will destroy the samples after three days of confirming that no additional tests are required.

Teen girls will be asked to provide a code word when they submit their urine sample for testing. This code word will be unique to them and something they will remember. This code word will be described to the teen girls as being: a word that only they will know and that other family members will not know; a word that the participant will be sure to remember; and a word that they will not share with anybody else. If teen girls ask for guidance in choosing a code word, we will suggest that teen girls might use the name of their favorite actor, musician, or favorite book. We will stress that teen girls are not to share their code word with their caregiver or parent/guardian and that the word should not be a word that is easily identifiable by anyone else. This code word, in addition to teen girls’ full name and date of birth, will then be used to verify identity and as an extra precaution to maintain confidentiality when disclosing STI test results.

UI Health’s Laboratory will screen the urine for STIs. The UI Health Lab will add the participant’s STI test results directly to their medical record in EPIC using their MRN. The CCTS Biomedical Informatics team will enable the Clinical Data Interoperability Services (CDIS) Module within our REDCap project to directly access this data from the Epic EHR. The CDIS Module is maintained by the REDCap development team and uses Fast Healthcare Interoperability Resources (FHIR) web service inside of an EHR which can pull structured data into REDCap using OAuth2 authorization. The CCTS team will enable this module and map the necessary lab data elements for the study team. When the study team needs updated data, they will refresh the data within REDCap for the enrolled participants using their MRN. The teen consent/assent form and the parental permission form include HIPAA authorization language and clearly state that the STI test results will be added to both the medical record and linked to identifiers in REDCap (referred to as the “secure encrypted server” in our consent forms).

A research team member will call the teen girl to disclose their STI test results as soon as they are received (see STI Notification Script). They will request the teen girl’s code word in addition to their full name and date of birth to confirm they are speaking to the right person before sharing the results. Participants will not have access to their results in the MyChart feature of EPIC to protect participants from having their parents/guardians view their results.

We are required to disclose positive STI results to the participant's local Department of Health. The report is expected to include what STI the participant was diagnosed with, the date the positive test was collected and the participant's name, address, phone number, date of birth, age, sex, race, and ethnicity. We disclose the above procedures in the consent/assent form for teen girls and the parental permission form. We inserted HIPAA authorization language into the teen girl consent/assent form and the parental permission form to further clarify what information we may be disclosing to the Department of Health. This disclosure is compelled by state law and is not a breach of confidentiality. Identifiable STI test result data will not be shared with our funding agency.

Teens will be offered STI treatment from a UI Health adolescent medicine physician or they can choose to receive treatment elsewhere (e.g., from their doctor). If the teen girl tests positive and chooses to get treated by the UI Health adolescent medicine physician, our UIC research team will work with the UI Health adolescent medicine physician to schedule the teen for an appointment. The UI Health adolescent medicine physician or their associates will have access to subjects’ medical records in EPIC. Additional details about standard clinical practice procedures for the STI treatment which are not part of this research are in Appendix 1.

For teen girls receiving an indeterminate result and who need to be retested or girls electing to receive treatment at UI Health who need help getting a CTA pass, we will transmit $5 to the participant via electronic cash transfer for the purchase of a 1-day pass. Participants can also transport themselves.

The UI Health adolescent medicine physician will add information about the STI treatment administered and the date(s) of service to the participant’s medical record in EPIC. The UIC research team will access the medical record to obtain this information and will transfer the information to the research participant’s record in REDCap so that all the participant’s testing and treatment information are stored together in a secure place.

For teen girls who choose to receive treatment elsewhere, our UIC research team will call them to request the following information: the name of the doctor where the participant sought STI treatment, the STI treatment received (we will ask them to describe the treatment if they do not know the name of the treatment- e.g., pill, shot), and the date(s) of service (see Script for STI Treatment on Own). The UIC research team will store this information in REDCap.

The UIC research team requires this information about STI treatment administered for our analysis of intervention effects on STI incidence; knowing whether research participants were cured of the STI(s) they test positive for will help us determine whether the intervention conditions have an effect on STI incidence. We disclose in the consent forms that teen girls are welcome to decline STI treatment or decline to answer any questions about their STI treatment and continue to participate in the research. The STI testing and treatment procedures detailed above have been followed over many years in other similar studies without any adverse events.

- - 1. WORKSHOP DELIVERY: Workshops will be two-days long, usually conducted on Saturday from 9am – 3pm and on Sunday from 9am – 1pm. Upon arrival to the CBO for a workshop, participants will check in with research staff, who will confirm baseline self-assessment surveys are complete and collect urine specimens according to procedures described in 6.2.9. Participants will be asked to finish any incomplete self-assessment surveys using either their cell phones or laptops/tablets provided by the research staff.

To avoid attendance bias, we will randomize all dyads in attendance to either the IMAGE or the FUEL workshop on the morning of workshop day one, using a 1:1 randomization ratio. Workshops will be held with at least three and up to eight dyads. If two dyads or fewer show up on workshop day one, the workshop will be canceled, and those dyads will be scheduled for another upcoming workshop date.

Breakfast and lunch will be provided on both days of the workshop.

Teen girls and caregivers will be asked to complete brief REDCap surveys about their experiences with the workshop’s implementation (see Participant Implementation Survey). Immediately after completing each workshop session, they will complete a very brief survey about the session. Additionally, after completing the full set of workshop sessions they have been randomized to, they will complete another survey about their experiences with the workshop as a whole. Teen girls and caregivers will complete these surveys in separate rooms using their cell phones or laptops/tablets provided by the research staff. Participants will go through the surveys on their own. UIC research staff will remain in the room to answer any questions.

- - 1. At the conclusion of workshop day 2, teen girls and male caregivers will each receive up to $125 total for the following completed activities: $80 for completion of 80% of baseline self-assessment questions and attendance of workshop day 1, and $45 for attendance at workshop day 2, in either cash or via electronic cash transfer (e.g., CashApp) according to the preference of the participant. If a participant completes the baseline self-assessments and workshop day 1 but does not come back for day 2, they will be compensated remotely at the conclusion of workshop day 2 via electronic cash transfer for $80 total.

If a participant completes the baseline self-assessment survey but does not attend their scheduled workshop, they may attend another workshop within 6 weeks, at which time they will be compensated for activities completed as above. If they are unable to attend a scheduled workshop within that time frame, they will not be compensated for survey completion; however, they can retake their baseline assessment and attend another workshop in the future if they wish, at which point they will be compensated up to $125 for activities completed.

Consent forms will state that participation in the study includes completing 80% of the self-assessment survey questions and attending workshop day 1; that their participation is completely voluntary, meaning that it is 100% their decision to participate; and that they may withdraw from the study at any time.

- - 1. Caregivers will be asked to complete remote follow-up self-assessment surveys in REDCap at 6- and 12-months post-workshop, using the same procedures as at baseline. Caregivers will receive $70 at 6-months and $75 at 12-months; compensation for male caregivers at follow-up will be based on self-assessment completion and offered remotely via electronic cash transfer (e.g. CashApp).
    2. Teen girls will be asked to complete 1) remote follow-up self-assessment surveys in REDCap at 6- and 12-months post-workshop, using the same procedures as at baseline and 2) additional STI testing at 6- and 12- months, using the same procedures as at baseline. Research staff may schedule a time to meet teen girls at CBOs to conduct STI assessment; staff may also schedule “open hours” at the CBOs where teen girls may stop by at their convenience to provide their specimen. If it is more convenient for teen girls, they may also meet staff on campus at UIC. To overcome transportation barriers, we will provide transportation to/from the CBO to obtain specimens from the teen girls. Teens will receive $70 at 6-months and $75 at 12-months; compensation for teen girls at follow-up will be based on self-assessment completion, and offered in either cash provided at the time of collecting the STI specimen, or if the STI specimen is not collected within data collection window, remotely via electronic cash transfer (e.g. CashApp).

**Aim 2 procedures in chronological order** are as follows:

- - 1. The study team will work with existing contacts at participating CBOs (see letters of support) to recruit approximately 10 CBO personnel to participate. Emails will be sent from study staff to establish CBO contacts (see attached recruitment email). Interested participants can submit their contact information privately via a secure REDCap link or call/text/email study staff.
    2. Study staff will follow up with interested participants to schedule a time to explain the project in more detail, answer questions, obtain consent, and conduct the initial semi-structured in-depth interviews (in person, by phone, or via Zoom).
    3. At the scheduled, private interview session, a study team member will explain the research study and obtain informed consent using REDCap electronic consent procedures.
    4. After consent is obtained, staff will conduct the participant interview, following the interview guide. The interview will be audio recorded. After the interview, the participant will be asked to complete secure surveys in REDCap. The interview and survey together are expected to take between 45-60 minutes.
    5. At the conclusion of the interview and survey, participants will receive a $25 e-gift card (e.g., Visa or Amazon).
    6. After the first IMAGE group is conducted at the participant’s CBO, we will reach out to schedule a second interview, using the same procedures as the first interview, using the Interview 2: Rollout Phase questions. Participants will be asked to complete secure surveys in REDCap and receive a $25 e-gift card (e.g., Visa or Amazon), as they did for the first interview and survey.
    7. After implementing IMAGE at the CBO, we will reach out to schedule a third and final interview session using the same procedures as the first and second interviews. Participants will be asked questions under the Interview 3: Sustainability Phase.
    8. If a participant is employed by the CBO after the first or second round of interviews are completed, they may be invited to participate in the study via email and consent obtained as described in steps 1-3 above but asked only to complete interviews 2 and/or 3, depending on the implementation phase in which the CBO employee is hired.
  1. Aim 1: Self-assessment surveys will be completed by both teen girls and caregivers. STI testing will be completed by teen girls via a urine specimen.

Aim 2: Interviews will be audio recorded and transcribed, and notes will be taken. In addition, the participant will be asked to complete a survey in REDCap.

- 1. There are no plans for long-term follow up after all research related procedures are complete.
  2. Aim 1: Survey questionnaires will assess the following constructs, per the attached quantitative measures:
  - Demographics
  - Legal history
  - Alcohol/substance use history
  - Sexual behaviors
  - Sexual communication history
  - HIV/STI knowledge, attitudes and beliefs
  - Emotion regulation
  - Coping with adverse life events
  - Condom self-efficacy
  - Sexual communication
  - Family risk and protection
  - Gendered racial socialization
  - Risk, adultification, and messaging
  - Stereotyping
  - Sexual objectification
  - Everyday discrimination
  - Justification of violence
  - Skin color satisfaction scale
  - Appearance and sexual decision-making
  - Caregiver-girl relationship and attachment
  - Brief resilience scale

Aim 2: The source records that will be used to collect data about subjects include the attached interview guides and the following attached quantitative measures in REDCap:

- - Demographic questions
  - ORIC
  - Organization Climate
  - Pre/Post Implementation Items (reach, adoption, implementation maintenance, acceptability, appropriateness, feasibility, and sustainability).

# Data Collection and Retention*

Data will not be banked for future use.

# Sharing of Results with Subjects*

# Study results will not be shared with subjects. Individual STI testing results will be shared with teen girls, using procedures outlined in section 6.2.

# Study Timelines*

Aim 1 will consist of 1) remote self-assessment surveys for all participants, which will take about an hour each to complete, at baseline, 6-months, and 12-months, and 2) a two-day workshop intervention described in section 5.0 and section 6.2. Girls in Aim 1 will also provide urine specimens for STI testing at baseline on workshop day 1, and again at 6-months and 12-months.

Aim 2 will consist of three separate interview sessions that will take between 45-60 minutes each. These three sessions will take place over the course of the implementation of IMAGE at the CBO, before, during, and after the implementation of IMAGE at the study site, which is expected to take between 1-3 years.

# Subject Population*

Aim 1: Teen girls must a) be 14-18 years old; b) self-identify as African American, Black, or mixed race teen girl; c) speak English since measures are not normed for other languages; and d) identify an eligible male caregiver to participate in the study.

Male caregivers must a) be >= 18 years old; b) self-identify as African American, Black, or mixed race; c) speak English since measures are not normed for other languages; d) be a current male caregiver to a Black teen girl 14-18 years old; and e) the teen girl’s legal guardian must agree to the male caregiver’s participation. Caregivers will be defined as men (fathers, grandfathers, uncles, brothers, cousins, etc.) whom teen girls report play a central role in their care and upbringing.

Aim 2: CBO directors and CBO IMAGE liaisons at participating CBOs, aged 18 or older, will be invited to participate in the study. Participants will be excluded if they cannot understand the consent process, are not employed at a partnering CBO, or cannot read and speak English.

# Vulnerable Populations*

Aim 1 of this study will involve 14-17 year old teen girls who have not attained the legal age for consent to research.

Aim 2 of this study will not involve vulnerable populations (e.g., minor children). Women who are pregnant may be participants in the research as CBO directors and/or liaisons, but they will not be actively recruited for participation, nor will they be excluded for participation based on this condition.

# Number of Subjects

The total # of research participants across both study arms is 630.

Aim 1: We will enroll teen girls (n=300) and their male caregivers (n=300).

Aim 2: We will initially enroll CBO directors (n=5) and CBO IMAGE liaisons (n=5) for a total 10 CBO personnel. If participants leave the organization, we will recruit additional CBO personnel to complete the second or third interview; recruiting no more than 30 participants total (up to 15 directors and up to 15 liaisons).

# Recruitment Methods

Aim 1: UIC research staff and/or CBO staff will conduct a variety of outreach and recruitment activities, including:

- posting recruitment flyers at the CBOs or other community sites.
- sharing recruitment flyers via social media platforms, websites, or listservs. Social media platforms will only be used for passive recruitment, directing interested participants to the REDCap Registration Form (see #2) – social media will not be used to directly interact with interested participants.
- approaching families in person to share the flyer, using the Approach Script.
- distributing flyers at relevant outreach events held either in person or in presentations over video conferencing platforms (e.g., Juneteenth, Bold expression festivals, community events, etc.) – Flyers will be shared via Zoom using visual presentation of the flyer on a shared screen.
- distributing flyers to participants who complete workshops, encouraging them to tell other families about the program.

If interested participants request that CBO staff give their contact information to UIC researchers, CBO staff will transmit contact information to the UIC research team via the Registration Form. UIC research staff will only call families who have entered their contact information in the Registration Form.

Interested participants can either 1) call/text/email research staff directly using contact information on the flyer or 2) access a QR code on the flyer that links to a secure REDCap Registration Form where they can enter their name, phone number, email address, and preferred time of contact. Caregivers who complete the REDCap registration form will also be asked to provide the name and contact information for a teen girl they think might be interested in participating. Teen girls who complete the form will be asked to provide the name and contact information of a caregiver they might want to invite to participate, as well as their legal guardian. The REDCap registration form will state that a parent or guardian’s permission is required for minors (14-17 years).

Research staff will follow up with potential teen girl participants separately from guardians or caregivers to conduct the screening, to obtain consent/assent with written electronic signature via the e-consent module in REDCap, and to confirm the contact information of legal guardians and/or participating caregivers.

Both male caregivers and teen girls will receive up to $125 at baseline, $70 at 6-months, and $75 at 12-months, in cash or via electronic cash transfer (e.g. CashApp), as described in detail in section 6.2. Note that although participants may receive payments up to $270, they will never receive more than $200 within a calendar year; tax identification information will not be collected from subjects.

Aim 2: The study team will work with existing contacts at participating CBOs (see letters of support) to recruit CBO personnel to participate. Emails will be sent from study staff to establish CBO contacts (see attached recruitment email). Interested participants can submit their contact information privately via a secure REDCap link or call/text/email study staff. Study staff will follow up with interested participants to schedule a time to explain the project in more detail, answer questions, and obtain consent (in person, by phone, or via Zoom).

Participants will receive a $25 e-gift card (e.g., Visa or Amazon) for each of three completed interviews and surveys for a total compensation up to $75.

# Withdrawal of Subjects*

Aim 1: An investigator may discontinue a participant from the study if they exhibit disruptive behavior during workshops or if continued participation in the study would put themselves or others at risk of harm; or if the participant meets an exclusion criterion (either newly developed or not previously recognized) that precludes further study participation.

Participants are free to fully withdraw from participation in the study at any time upon request, by contacting research staff by phone, email, or letter as directed on the consent form.

Aim 2: There are no anticipated circumstances under which subjects will be withdrawn from the research. Subjects may withdraw from the study by simply declining to participate in follow- up interview sessions.

# Risks to Subjects*

- 1. The risks associated with this study are minimal and mainly concern (a) a potential breach of privacy (i.e., others may find out that the individual participated in the research), b) a potential breach of confidentiality (i.e., others may find out what the individual said or disclosed during the research), (c) discomfort with the assessment questions and intervention materials, and (d) receipt of the incorrect STI result (applicable to teen girls in Aim 1 only).

Because staff from the CBOs may be onsite when workshops are conducted or when teen girls meet with research staff to provide STI samples, CBO staff may know whether or not an individual decided to participate in the study. While researchers will ask others in the workshop group to respect each other’s privacy, and not repeat what is said to others outside of the group, this confidentiality cannot be guaranteed. If a subject discloses abuse or neglect, we are mandated to report these concerns and will have to inform the appropriate authorities. Participants will be clearly informed of these exceptions to confidentiality during the informed consent process.

Teen girls and caregivers may feel uncomfortable with answering the self-assessment questions, they may get upset when sensitive topics come up during the workshop sessions, and/or they may experience discomfort talking to each other about difficult topics. Of note, most of the measures have been used extensively in clinical research with no known adverse effects.

For teen girls, there is a chance that their STI results could be incorrect. If this happens, there is a chance that a teen girl could be treated for an STI even if they are negative, or they could not receive treatment even if they are positive.

- 1. Procedures to minimize risk of breach of privacy and confidentiality, as well as procedures to minimize risk of incorrect STI result are both described in section 6.2 (e.g. code word verification for STI results; dual verification of STI results) and 17.2 (e.g. data security and handling).

To minimize the risk of discomfort, we will ensure that all participants are aware that their participation is completely voluntary, and that their decision to participate or not will not affect their relationship with the university in any way. We will explain to participants that if they feel uncomfortable or uneasy at any point during their participation, or if they simply change their mind, they are free to withdraw from the study at any time. All research staff will participate in the CITI ethics training, attend initial training sessions with the PIs, and receive ongoing supervision in areas related to ethics, confidentiality protection, and other topics of human participant protection.

Finally, our research team has over 20 years of experience conducting workshops with families where sensitive topics are discussed. We do not expect participants to spontaneously disclose abuse, violence, or significant risk of harm to self or others. However, if this does occur, trained UIC research staff will conduct clinical incident procedures to 1) assess participants’ level of risk by collecting detailed information about the possible clinical event, 2) create a safety plan with the participant, including if there is the potential for immediate serious harm to the self or others, and to make relevant referrals (e.g., for local mental health services) as necessary. In cases of imminent risk, the PI, a Registered Nurse, is notified for immediate consultation and follow-up. If a subject is in danger of hurting themselves or others, we will work with the participant to arrange for their safety (e.g., hospitalization). If a subject discloses abuse, violence, or significant risk of harm to self or others, we are mandated to report these concerns and will have to inform the appropriate authorities. Participants will be clearly informed of these exceptions to confidentiality during the informed consent process and at the beginning of the workshops; clarifying the limits of confidentiality allows participants to make fully informed decisions about whether to participate. Reporting will be done as appropriate to the situation and the legal statutes, including reporting to child protection agencies or other appropriate agencies. Of note, these clinical procedures have been used by the research team for over 20 years with no known harmful effects.

# Potential Benefits to Subjects*

There is no direct benefit to the participant for participation in the study, although we hope that teen girls and their caregivers will enjoy the workshop intervention. Teen girls may also derive benefit from receiving STI testing and treatment, as needed.

# Data Management* and Confidentiality

- 1. Survey data will be analyzed using descriptive statistics and by calculating an aggregated mean total score and means for each CBO. Using a continuous and iterative process will identify the contextual factors (events or statements) from interviews to document what facilitates or acts as barriers to implementing IMAGE. To begin the qualitative analysis, study staff trained by the PI will immerse themselves in the data by reading and re-reading the transcripts and noting the perceptions of the interviewees. Using the Dedoose (Version 8.2.32) and following an approach described by Miles and Huberman a set of initial codes grouped into broad domains reflecting the interview guide and EPIS framework (i.e., groups of related codes) will be developed. EPIS constructs will include Outer context – relationships between entities, including governments and funders; Inner context – the structure of CBOs, culture, networks, communication, climate, and readiness for implementation; and Bridging factors –the relationship between CBOs and UIC and Innovation – CBO and IMAGE fit and sustainability. Staff will open code two transcripts at a time to refine codes into a preliminary codebook with clear operational definitions. Interviews will be separately coded, and then they will consult with the PI and other team members to review discrepancies, refine code definitions, and recode until intercoder reliability exceeds 85%. Final codes will be compiled in the master codebook and applied to coding all qualitative data. The research team will collaboratively analyze results, discuss codes, categories, and themes generated, and resolve discrepancies through discussions. This iterative process will allow us to identify the most salient contextual factors (events or statements) from observations (study notes) and interviews and document implementation barriers and facilitators (e.g., challenges, resolutions, impacts of champions, leadership, etc.). Final categories and themes will guide any necessary revisions of implementation procedures and the CBO’s implementation plan. We expect to identify shared and unique experiences from each CBO. We will triangulate qualitative data with quantitative measures related to treatment delivery and receipt of treatment. Together these data will provide insight from all perspectives on program success and potential future integration and sustainability of IMAGE by CBOs. Measures supporting rigor and trustworthiness in qualitative research include a detailed audit trail, study notes, and reflexivity notes. Each audio recording will be transcribed and checked for accuracy.
  2. Data Security and Handling.

Coded data and REDCap. Participants will be assigned in REDCap a unique research id that will be used in lieu of individual identifiers on all data collected from participants (including via survey assessments, STI results, and qualitative interviews), except for urine specimens which will be labeled only with the MRN and date of birth. Unique research ids will not be comprised of birthdates, initials, or any other identifying information that could reveal the identity of the participants. Unique research ids will be securely stored with individual identifiers (i.e., participants’ name, phone number, email, date of birth, MRN, and address) in REDCap, making REDCap an inherent master list kept separately from coded data exported from REDCap. REDCap will link participants to their data over the course of the longitudinal study, and study personnel will only access identifiable information in REDCap on an as needed basis to adequately perform their duties. No one outside the IRB-approved study team will have access to REDCap or to identifiers of any kind.

Secure data storage. STI testing and treatment results, survey data, identifiers, and unique research ids will be securely stored in REDCap. Coded data exported from REDCap without identifiers, interview recordings, interview notes, and transcripts will be stored on a password-protected subdirectory of a secure server in the Department of Medicine at UIC. All study computers will be encrypted. Any hard copies of interview notes will 1) be scanned for digital storage on a password-protected subdirectory of a secure server at UIC and then 2) be destroyed. These notes will not contain identifiers; no other hard copies will be used for data collection.

REDCap. The REDCap server is behind a firewall, has virus protection, uses Secure Socket Layer authentication to encrypt communication between a user and the server, and has been configured to meet campus HIPAA rules. REDCap has secure authentication password login and sophisticated user rights controls that can restrict access as needed user by user to certain REDCap functions but not others (e.g., allowing data exports or not, viewing summary statistics and charts, adding or editing records, etc.). REDCap incorporates an electronic audit trail to show changes to data after original entry including the date/time and user making the change.

Staff training. All study personnel will complete extensive training before they are granted access to identifying information. They will complete the Human Subjects Training required by the University of Illinois at Chicago, which complies with federal guidelines delineated in 45 CFR Part 46. Personnel will participate in training with the Principal Investigator and/or the Project Director regarding data safety, confidentiality of participants, limits of confidentiality, and proper administration of the study protocol.

Audio-recordings. Interviews will be digitally audio recorded using either Zoom recording functionality or a secure audio recording device (e.g., an encrypted University-owned laptop computer or portable audio recorder with a microphone). If recording using Zoom, both audio and video files are automatically recorded, due to Zoom functionality. Following the interview, the Zoom video file will be immediately destroyed, and the audio-only file will be retained for study purposes. Regardless of recording mechanism, audio files will be uploaded immediately to a password-protected folder on a secure server and deleted from all devices. Study staff will maintain confidentiality during qualitative data collection and the transcription process. During interviews, the participant’s code number will be used to identify the speaker, and study staff will remind the participant before the interview begins not to refer to themselves by name.

Audio-transcription. Audio-files will be transcribed by Rev.com; audio files and transcriptions will stored by and transmitted to and from Rev.com using 128-bit SSL encryption, the highest level of security available. Transcribed files are password protected and sent back to the research team via secured electronic files; confidentiality agreements are also obtained. Once the recording has been transcribed and verified, any identifying information, besides the participant’s code number, will be deleted from the transcript and the recording will be destroyed.

Disseminated data will not identify specific participants. Manuscripts and other written reports will only display data in aggregate. Datasets will be de-identified before being shared outside the approved research team. Identifiers will be destroyed at the conclusion of data analysis.

A Certificate of Confidentiality will be issued by the federal government that will be maintained through the life of the research project. This protects the data from subpoena and thus prevents the data from being used against the participant. Study staff will inform participants that criminal behavior, i.e., drug use, is not reported to authorities, and that the security of this information is protected by the Certificate of Confidentiality. Study staff will inform study participants of the limits of confidentiality during the consent process. Specifically, study staff will warn participants that state laws mandate reporting of abuse and/or neglect of children or elders, and that threat of harm to self or others requires intervention by clinical staff.

# Provisions to Protect the Privacy Interests of Subjects

- 1. If participants are interested in the study, they may visit a secure REDCap link to enter their contact information or they can privately reach out to study staff via call/text/email. In this way, participants can control when, how, and whether they initially contact researchers. In addition, all contact information will be securely stored in REDCap, and will not be shared outside the research team.
  2. During recruitment, consent, the self-assessment surveys and the interview process study staff and/or research materials will emphasize that the information is being collected for research purposes only and will not be shared outside the research team. CBO staff in Aim 2 will be assured that their information will not be shared with their employer or other parties. Participants will be assured that they are free to decide not to answer any question that may make them uncomfortable.
  3. The research team will only access information that is provided directly from participants; either from participants initiating contact with study staff, or during interviews and assessments conducted after participants provide their consent.

# Economic Burden to Subjects

- 1. There are no costs to study participation other than the potential cost of transportation to CBOs for workshops, to CBOs for follow-up assessments, and/or to receive STI treatment if needed.

# Consent Process

Aim 1: Using the Screening and Consent Script, research staff will follow up with potential teen girl participants separately from guardians or caregivers to conduct the screening, to obtain consent/assent with written electronic signature via the e-consent module in REDCap, and to confirm the contact information of legal guardians and/or participating caregivers. We will obtain teen girls’ assent/consent before we contact caregivers, because the teen girls’ refusal to participate will take precedence over consent or permission by male caregivers and legal guardians.

After a teen girl’s assent or consent has been obtained, research staff (not CBO staff) will follow up with caregivers and/or guardians as appropriate to obtain consent and/or permission with written electronic signature via the e-consent module in REDCap. If the minor teen girl would like to invite a caregiver to participate who is not the guardian, UIC research staff will share the name of this person with the guardian when permission is obtained. If the teen girl later changes her mind about which caregiver she would like to invite, UIC research staff will re-contact the guardian for permission and will complete the addendum included in the permission form.

Research staff may conduct screening and consent procedures by phone, via UIC Zoom, or in person in a private space. All consent procedures will be conducted via REDCap, even if conducted in person. Links to consent forms in REDCap will either be emailed or texted to the participant, according to their preference, or study tablets or laptops may be used if consent is conducted in person. No paper forms will be used. Staff will go over consent forms with participants and confirm in REDCap that the study was explained fully, the study participant could understand the information provided, and the study participant was given ample opportunity to ask questions. REDCap will be configured to automatically send signed consent/assent/permission forms to participants via email or text according to their preference. No study-related procedures will be completed prior to obtaining informed consent.

The e-consent module in REDCap will display consent forms stamped by the IRB as images (e.g. as a PDF), and will collect signatures from participants within REDCap fields. Therefore, to avoid confusion and redundancy, forms submitted for approval to the IRB will not include lines for signature, date, and printed name of participants, nor signature, date, and printed name of staff obtaining consent, , as all of these will be collected directly within REDCap.

Aim 2: Upon expressing interest in the study and scheduling a private interview, research candidates will be provided a copy of the consent form to review contained in the scheduling email. Prior to the interview, study staff will review the consent form with potential participants and answer any questions the participant may have. Study staff will send a REDCap link for the participant to sign the consent form using the REDCap e-consent module. During the consent process, care will be taken to ensure that the liaisons and directors are willing participants. We will highlight the right to decline and that participation is voluntary. The participants are encouraged to ask questions throughout this process. Participants are informed again that they are free to withdraw at any time.

# Setting

All research activities (screening, consent, surveys, STI testing, interviews) will take place at the CBOs, on campus at UIC as needed, or remotely via telephone or UIC Zoom. As of March 2024, we have engaged four CBOs, listed below, that are interested in implementing IMAGE at their site. Over time, we may partner with additional CBOs not listed below.

# Resources Available

**We are partnering with Community-based organizations (CBOs) for this project**. As of March 2024, we have engaged four CBOs that serve Black teen girls and male caregivers as partners in this study. Each CBO seeks to reduce health disparities by providing programming, social services, and care to address community resource needs and promote health and behavior change. The CBOs are located in Chicago neighborhoods highly impacted by structural factors (i.e., racism, poverty, crime, incarceration, and urban decay) implicated in STIs. They will serve as the sites for our RCT with Black male caregivers and teen girls. Over time, we may partner with additional CBOs not listed below.

Figure 1 provides an overview of where each community-based organization is located.


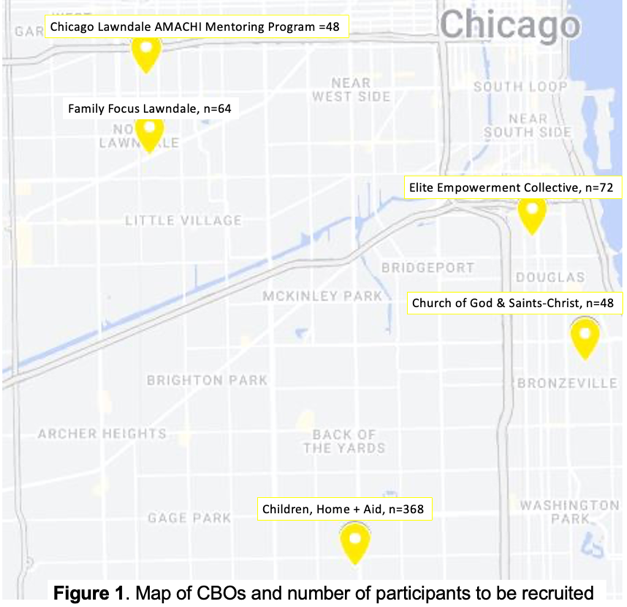


**Chicago Lawndale AMACHI Mentoring Program (LAMP) (Green).** LAMP provides mentoring to children 5-24 years old impacted by incarceration and delivers programming focused on academic achievement, decision-making, social and emotional stability, and community pride in North Lawndale, Chicago. Dr. Crooks has been volunteering with LAMP over the past year facilitating monthly health education workshops (i.e., sexual health, mental health, bullying, self-esteem) workshops for Black teen girls. Dr. Green the director of LAMP, is supportive of the proposed study (see Letter of Support) and is currently facilitating recruitment for a different study. Since joining UIC in August 2019, the PI has developed collaborations with LAMP. LAMP has been Dr. Crooks longstanding community partner, as they have supported Dr. Crooks research by providing space for data collection at their facility and aiding with recruitment. Dr. Crooks conducted her pilot of IMAGE at the LAMP facility this summer. Dr. Green and the LAMP staff will consult throughout the study. The proposed work will strengthen partnership development and will allow for a long-term trusted relationship with Black Chicago communities.

**Family Focus Lawndale (FFL).** FFL is a not-for-profit family support center serving those in the North Lawndale community of Chicago. Their mission is to promote the well-being of children from birth by supporting and strengthening their families in and with their community. FFL is a pioneering family support organization founded in Chicago in 1976. FFL offers innovative, community-based programs that help parents, grandparents, and foster and adoptive parents, gain confidence and competence as the primary educators of their children. Family Focus has eleven centers in Chicago and surrounding communities. FFL serves 510 Black families in the North Lawndale neighborhood.

**Brightpoint (formerly Children Home and Aid).** Brightpoint is a leading child and family service agency in Illinois serving nearly 30,000 (23,000 Black) families each year in over 65 counties. They are an agency that puts families at the center of every decision and believes that communities where they all work, play, and live together, can be strengthened through data-informed, collaborative, and preventative solutions. Their strategic plan includes an emphasis on prevention, family as a critical asset to child well-being and an intentional focus on racial and social equity. We will specifically be targeting the Englewood neighborhood.

**Elite Empowerment Collective (EEC).** EEC is a non-profit organization that supports youth development through dance. Many of their youth are Black teen girls aged 14-18 years old. Dr. Crooks previously collaborated with EEC on another project, recruiting Black male caregivers for qualitative interviews in the early phases of IMAGE intervention development. EEC serves over 300 Black families and youth in the Wrightwood/Bronzeville area.

**Appendix 1:** **STI Treatment Procedures**

*STI treatment is offered outside the scope of research, as a service to teen participants who test positive for an STI and elect to receive treatment at UI Health. UI Health requires that treatment services paid for by funds from a research study be documented in the IRB protocol, even if those procedures are strictly conducted by UI Health according to standard clinical practice and are not determined or performed by the non-clinical research team. The purpose of this appendix is to document the clinical STI treatment procedures that may be carried out by UI Health.*

*As described elsewhere in the protocol, our study team will 1) notify teens who test positive for an STI that they may receive treatment for free at UI Health, 2) support them with accessing the treatment, and 3) record which treatment they received and on which date(s); our study team will otherwise not be involved with the provision of STI treatment to teens, and no other information from the following procedures will be abstracted into our research records, reviewed, or tracked in any way. These procedures are explained to participants in the teen assent/consent form and parent/guardian permission form.*

When a participant meets with the UI Health adolescent medical doctor/physician, a pregnancy test will be administered. This is required to determine the STI treatment that will be administered, since some forms of STI treatment cannot be given to individuals who are pregnant. STI treatment will be given as follows:

- Chlamydia:
  - Standard treatment: Azithromycin 1g (single dose; tablets).
  - Doxycycline 100 mg (7-day dose; caps) for those with contraindications.
- Trichonomiasis:
  - Standard treatment: Tinidazole 2g (single dose; tablets).
  - Metronidazole 500mg (7-day dose; tabs) for those with contraindications.
- Gonorrhea:
  - Ceftriaxone 500mg (single dose injection), plus a dilutant (lidocaine 1% or sterile water 5mL vial)

The UI Health physician’s team will route the prescription to the pharmacy. All medications will be tubed to the clinic. The participant will wait for the medication in the clinic. When administering treatment for chlamydia or trichomoniasis, for single doses, the UI Health physician will observe oral medications. For 7-day doses, the participant will take the medication home. When administering treatment for gonorrhea, a member of the UI Health physician’s team will administer the injection.

The UI Health physician will ask teen participants who test positive for chlamydia and/or trichomoniasis if they would like to receive expedited partner therapy (EPT), which refers to the clinical practice of treating the sex partners of patients diagnosed with an STI by providing prescriptions or medications to the patient to take to his/her partner without the health care provider first examining the partner. If the teen wants partner treatment, the participant will need to provide the name of the partner when collecting the treatment. Partner treatment for chlamydia and/or trichomoniasis will be paid for by the study given that it is best practice to provide partner treatment when someone tests positive for an STI, but the research study will not access or record the identity of the partner.

Participants will be informed that partner treatment for gonorrhea will not be offered since it is given as an injection. Procedures for EPT will be exclusively managed by the UI Health physician and their team and are outside the scope of the research.
